# Healthy Sleep Practices for Consumers of Home Total Parenteral Nutrition: A Mixed-Methods Community-Based Participatory Study

**DOI:** 10.1016/j.cdnut.2024.102155

**Published:** 2024-04-05

**Authors:** Adline Rahmoune, Christine Spadola, Bethany Johnson, Steve McCarthy, John Winkelman, Charlene Compher, Marion Winkler, Hassan S Dashti

**Affiliations:** 1 Department of Anesthesia, Critical Care and Pain Medicine, Massachusetts General Hospital and Harvard Medical School, Boston, MA, United States; 2 School of Social Work, University of Texas, Arlington, TX, United States; 3 Transplant Unwrapped, Flower Mound, TX, United States; 4 Patient Author; 5 Department of Psychiatry, Massachusetts General Hospital and Harvard Medical School, Boston, MA, United States; 6 Biobehavioral Health Sciences Department, University of Pennsylvania School of Nursing, Philadelphia, PA, United States; 7 Department of Surgery, Rhode Island Hospital, Alpert Medical School of Brown University, Providence, RI, United States; 8 Division of Sleep Medicine, Harvard Medical School, Boston, MA, United States; 9 Division of Nutrition, Harvard Medical School, Boston, MA, United States

**Keywords:** Home parenteral nutrition, sleep, mixed-methods study, nutrition, quality of life, guidelines

## Abstract

**Background:**

Consumers of overnight home parenteral nutrition (HPN) often experience sleep disruption; however, existing healthy sleep recommendations are widely inapplicable to consumers.

**Objectives:**

The aim of this mixed-methods, community-based participatory research study was to develop tailored recommendations on healthy sleep practices for HPN consumers.

**Methods:**

The multipart study involved the following: *1)* an initial draft of sleep recommendations based on the evaluation of existing general sleep hygiene guidelines by an expert panel of clinicians and consumers with lived experience; *2)* semi-structured focus groups with consumers and clinicians; *3)* pre- and post-knowledge tests completed by consumers, and *4)* final approval of the recommendations by the expert panel.

**Results:**

The literature synthesis resulted in 51 recommendations evaluated for relevance for HPN consumers. Focus groups with 20 HPN consumers and clinicians contributed additional recommendations based on lived experience. Ultimately, the final resource included recommendations spanning 4 sections: getting ready for bed, preparing the bedroom for sleep, daytime behaviors, and overall strategies for better sleep. Of the 36 recommendations, 58% were derived from existing general sleep hygiene guidelines, and the remaining 42% addressed sleep challenges experienced uniquely by consumers, including nocturnal polyuria, noise/light from medical equipment, and infusion schedules. Knowledge tests completed by 10 additional consumers indicated a modest increase in sleep health knowledge.

**Conclusions:**

The curated healthy sleep resource tailored for HPN consumers was facilitated by a multidisciplinary expert panel, a strategic collaboration with members of the HPN community and their clinicians, and in partnership with patient advocacy and support organizations. The wide distribution of these resources may improve the overall well-being of HPN consumers.

## Introduction

Sleep is known to perform critical restorative functions and is necessary for overall health and well-being [1,2]. Insufficient sleep is a contemporary public health challenge with profound implications for physical and mental health [3], including adverse effects on daytime cognition, mood regulation, and cardiometabolic, neurologic, and immunologic functions [[4], [5], [6], [7]]. Among the leading factors contributing to inadequate sleep are chronic and acute illness, medication use, poor sleep hygiene, life stressors, and racial and socioeconomic inequities [8]. Consequently, emerging sleep guidelines and public health campaigns aim to educate people on best sleep practices to reinforce the central role of sleep on health [9]. These recommendations, often referred to as sleep hygiene guidelines, describe evidence-based lifestyle and environmental strategies that promote better sleep, including information on sleep schedules, bedroom environment, behavioral modifications, and dietary intake [10]. Sleep hygiene guidelines have been shown to be effective in promoting sleep across a range of populations [[11], [12], [13]]. As there is no “one-size-fits-all” for sleep recommendations, tailored sleep practices have been developed for clinical populations such as patients with insomnia and sleep apnea and, more recently, nonclinical vulnerable individuals, including children, adolescents, and night shift workers [[14], [15], [16], [17]].

Home parenteral nutrition (HPN) is the intravenous administration of nutrients at home for patients who cannot eat by mouth or adequately digest and/or absorb nutrients through their gastrointestinal tract and is regarded as one of the most complex home medical therapies [18]. Consumers of HPN range from acute (few months) to lifelong (>30 y) users and include patients living with short bowel syndrome, Crohn’s disease, and gastrointestinal dysmotility [19]. Infusions of HPN generally last 10–16 h and are cycled overnight by >80% of consumers to facilitate mobility during the day [20]. Overnight infusions of HPN are widely recognized by patients and their clinicians to disrupt sleep [21,22]. Patients attribute sleep disruption to nocturnal polyuria, infusion pumps, intravenous lines, and ostomy output [23].

Existing healthy sleep guidelines are widely inapplicable to HPN consumers as some elements of general sleep guidelines are impractical, insensitive, or irrelevant to this patient population. For example, existing guidelines assume that all people eat by mouth, recommend refraining from daytime napping, and encourage sleeping in a dark, quiet environment, which may not be entirely feasible for some HPN consumers. Current guidelines also fail to account for important challenges experienced by many consumers, including dealing with intravenous lines and medical equipment. The purpose of this mixed-methods, community-based participatory research study was to develop tailored recommendations on healthy sleep practices for consumers of HPN. The wide dissemination of this information in partnership with patient advocacy organizations will likely empower consumers and their clinicians with essential knowledge to prioritize healthy sleep practices.

## Methods

### Study design

**FIGURE 1:**
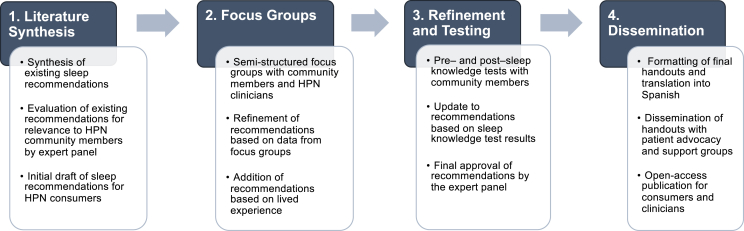
Flowchart of study approach and aims. HPN, home parenteral nutrition.

This was a mixed-methods, community-based participatory research study involving consumers of HPN (referred to here as *community members*), clinicians caring for HPN consumers, and a multidisciplinary expert panel. The study protocol was approved by the Mass General Brigham Institutional Review Board (protocol #2022P003373). The multipart study involved the following components to generate recommendations on healthy sleep practices uniquely tailored for HPN consumers: *1)* an initial draft of sleep recommendations based on existing general sleep hygiene recommendations evaluated by an expert panel; *2)* semi-structured focus groups with community members and clinicians to refine the initial draft; *3)* pre- and post-knowledge tests completed by HPN consumers to evaluate the updated draft; and *4)* final approval of the recommendations by the expert panel (Figure 1).

The multidisciplinary expert panel comprised a sleep scientist with expertise in mixed-methods research, a sleep medicine physician and scientist, 2 HPN clinicians with expertise in quantitative and qualitative nutrition support research, a nutrition expert, and 2 community representatives and advocates with extensive lived experience in HPN. Additional research participants in this study included community members and clinicians. To facilitate recruitment of research participants, the study was advertised at patient advocacy and support organizations and conferences (e.g., Oley Foundation and Transplant Unwrapped), social media interest groups (e.g., Facebook), and relevant clinics. Participants expressed interest through the Mass General Brigham clinical research website, Rally (https://rally.partners.org/). Potential participants responded to an online screener via Research Electronic Data Capture (REDCap) to assess eligibility. For community members, the inclusion criteria were as follows: adults (age 18–79 y), residing in the United States, currently infusing HPN overnight for ≥6 mo with no major changes, and able and willing to give consent and comply with study procedures. For clinicians caring for consumers of HPN, the inclusion criteria were as follows: adults (age 18–79 y), residing in the United States, currently caring for consumers of HPN who run infusions overnight.

Similar to other recent online qualitative research studies, the study was prone to “imposter participants” (i.e., fake participants). Indicators of imposters, as previously described [24], were carefully reviewed, including configurations of email addresses and vague responses to HPN-specific screening questions. Suspected participants were further contacted by telephone to corroborate screening responses. Participants who were deemed as potential imposters were excluded.

Eligible participants provided electronic consent before the start of study procedures. Basic demographic information, including age, gender, race, ethnicity, and employment status, was collected from eligible consumer participants. Further HPN-specific information was collected, including timing and duration of HPN infusions and quality of sleep patterns. From eligible clinician participants, information on the role of the clinician was collected. All research participants were offered up to $100 for their contribution to the project.

### Synthesis of recommendations

First, a literature review was conducted to synthesize existing sleep guidelines. Recommendations were compiled from governmental agencies (e.g., the Centers for Disease Control and Prevention, and the National Heart, Lung, and Blood Institute), foundations (e.g., the National Sleep Foundation), professional societies (e.g., the American Academy of Sleep Medicine), clinical practice guidelines, and peer-reviewed scientific articles. The following search terms were used in conjunction with sleep: “guidelines,” “resources,” “tips,” “recommendations,” “suggestions,” “treatments,” “interventions,” and “management.” Recommendations were then grouped by theme and target populations. Themes included consistency and routine, the bedroom environment, physical activity, intake before bed, behavioral strategies, medication interference, nocturnal polyuria, relaxation and comfort, and pharmacologic strategies (Supplementary Table 1). Target populations ranged from generally healthy adults to patient subgroups including those living with insomnia, sleep apnea, Restless Legs Syndrome, cancer or receiving chemotherapy treatment, nocturnal polyuria, or receiving nocturnal hemodialysis. The expert panel convened to assess each recommendation for the following: *1)* practicality and *2)* relevance to community members based on expert knowledge and lived experience. Items deemed impractical or irrelevant (i.e., conflicting or insensitive with standard HPN home care or impossible to adhere to with HPN) were excluded. The remaining strategies were consolidated to yield an initial draft of healthy sleep practices.

### Focus groups

Semi-structured focus groups were conducted separately with community members and clinicians to gain feedback on the initial draft, identify any potential gaps, and obtain further input from lived experience. Focus groups were selected for their format, which allows for the sharing of responses, the exchange of ideas, and the generation of discussion among participants [25,26]. Focus group guides were created with support from the Massachusetts General Brigham Qualitative and Mixed Methods Research Unit and revised in an iterative process with the expert panel (Supplemental Material 1, Supplemental Material 2). Briefly, community members were asked about their knowledge of general sleep hygiene, experiences with clinicians regarding sleep, strategies for coping with sleep challenges, and opinions on specific sleep recommendations, such as maintaining a consistent sleep routine and noise-masking infusion pumps. Similarly, clinicians were asked about their knowledge of sleep health, discussions with their patients on sleep, feedback on specific sleep recommendations from a clinical perspective, and potential gaps.

Each focus group lasted ≤1 h, was conducted remotely via an encrypted institutional Zoom meeting, and was led by 2 members of the study team (AR and HSD) using an IRB-approved focus group guide. The community focus groups were also moderated by a community representative from the expert panel (BJ and SM). Discussions were recorded and transcribed for analysis. The initial draft of the recommendations was subsequently revised based on the gathered data.

### Knowledge test

Additional community members were recruited to complete pre- and post-knowledge tests to further examine the refined sleep recommendations. First, participants completed a 30-item pre-knowledge test on general sleep health (Supplemental Material 3). Next, participants were presented with the drafted recommendations. For each recommendation, participants were asked to rate the usefulness to HPN consumers on a 4-point Likert scale and to provide any additional feedback. Lastly, participants completed a post-knowledge test. Participants were able to pause and return to the survey at a later time. Components of the knowledge test were administered electronically via REDCap surveys. Knowledge tests were self-paced and completed remotely and asynchronously by study participants. At the conclusion of the post-knowledge test, participants rated the overall usefulness of the information presented, the likelihood of adopting the recommendations, and the likelihood of sharing the resource with other HPN consumers on a 4-point Likert scale. Participants were also provided a free text option to share any additional feedback or comments on the resource.

The number of correct responses was compared between the pre- and post-knowledge tests. Additional feedback was evaluated. Items that were considered not useful (i.e., response options “somewhat unuseful” or “extremely unuseful”) by 40% of the participants were re-evaluated by the expert panel. An updated version of the recommendations was subsequently generated.

### Final version

The expert panel convened a final discussion of the recommendations. Each individual recommendation was voted on for inclusion [17]. Any final feedback from experts was discussed and incorporated in the final version of the recommendations. All finalized materials were then translated into Spanish by the Massachusetts General Hospital Translation Services **(**Supplemental Material 6**,** Supplemental Material 7**)** and formatted into a handout by the Massachusetts General Hospital Photo Lab, which provides creative solutions, including digital work, to help with graphic materials. The final English and Spanish versions were submitted for publication on the website and newsletter of patient advocacy and support groups including the Oley Foundation, the largest home nutrition support patient education and advocacy nonprofit organization [27,28].

## Results

### Literature synthesis

**FIGURE 2:**
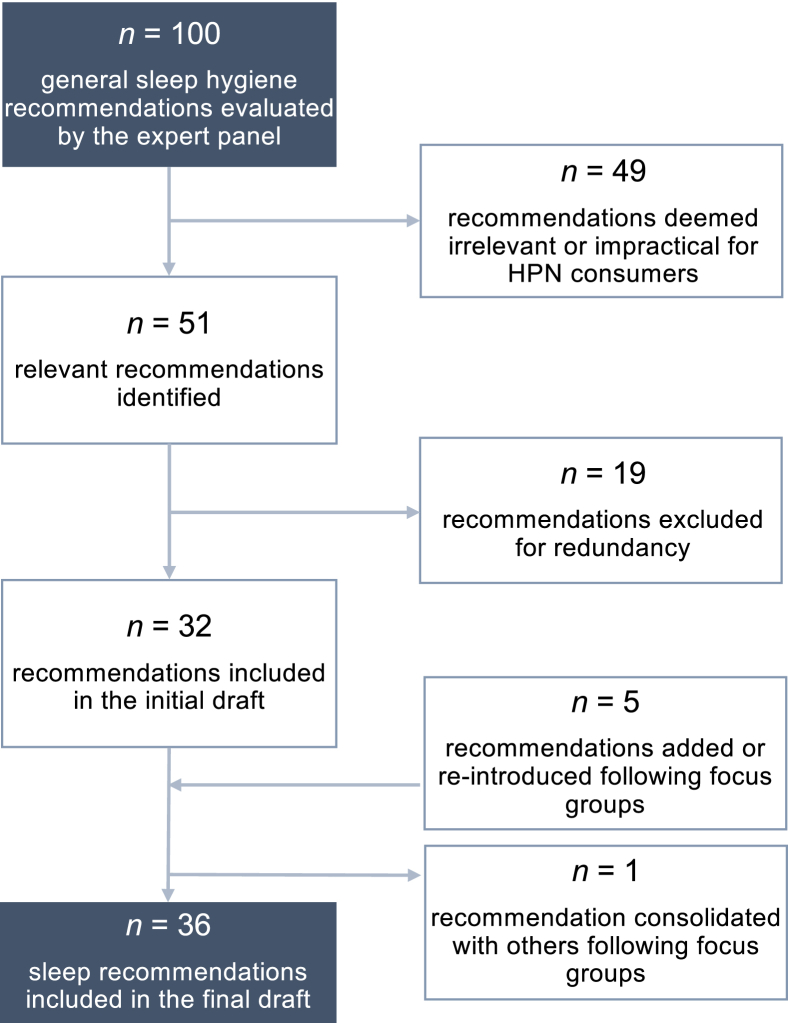
Flowchart of recommendations included in sleep health resource. HPN, home parenteral nutrition.

The literature review resulted in 144 compiled recommendations spanning the areas of sleep environment (exposure to light, noise, electronic devices; 22.9% of the identified recommendations), behavioral strategies for better sleep (stress reduction; 22.9%), dietary intake and physical activity (18.1%), sleep medications and medication side effects (16.0%), general sleep hygiene practices (sleep duration, sleep schedule consistency, time in bed; 13.9%), daytime napping (3.5%), and smoking cessation (2.8%) (Supplemental Table 1). Similar recommendations from different sources were consolidated resulting in 100 distinct recommendations. Of the remaining recommendations, 49 were deemed to be impractical or irrelevant (i.e., recommendations that were disease-specific). For example, recommendations pertaining to medication or supplementation use, except for melatonin, were excluded to limit potential drug–drug interactions. This resulted in 51 recommendations that were deemed relevant for HPN consumers. Recommendations with similar content, for example cognitive behavioral therapy and behavioral relaxation strategies, were further consolidated resulting in 32 recommendations (Figure 2).

### Enrollment of research participants

A total of 98 online responses were received from community members, of which 73 were deemed ineligible. Ineligible responses were excluded because of residing outside the United States (1 response), not providing contact information (2 responses), inadequate duration or timing of HPN (4 responses), and for likely being an imposter participant (66 responses). Of the 25 eligible responses, 19 completed the focus groups or sleep knowledge test, whereas the remaining 6 did not participate in the study. A total of 15 responses were received from interested clinicians to participate in the study, of which 4 responses were deemed as imposter participants, and the remaining 11 completed the clinicians’ focus groups.

### Focus groups

A total of 9 community members and 11 clinicians participated in a total of 2 community and 2 clinician focus groups, respectively. Demographic data of the focus group participants are presented in Table 1. The average age of participants (*n* = 9) in the community focus groups was 52.4 y (SD = 12.5) and 77.8% identified as female. Participants had been dependent on HPN for a median of 10 y (Q1, Q3 = 6, 35) and more than half (66.6%) cycled their infusions 7 nights a week. The most common indication for HPN was short bowel syndrome (66.7%), and 88.9% also ate by mouth. Discussions at the community focus groups included known HPN-related sleep challenges, including nocturnal polyuria, infusion pump alarms, infusion timing and schedules, and abdominal discomfort. Participants shared coping strategies drawn from lived experience. Community members emphasized the value of early but flexible sleep guidance and counseling.

**TABLE 1:** Demographic and nutrition characteristics of community member participants

	Focus group participants (*n* = 9)	Knowledge test participants (*n* = 10)	Total (*n* = 19)
**Age, y (mean, SD)**	52.4 (12.5)	39.8 (14.0)	45.8 (14.5)
**Gender, female (*n*, %)**	7 (77.8)	7 (70.0)	14 (73.7)
**Race (*n*, %)**			
White	9 (100)	9 (90.0)	18 (94.7)
Other	0 (0.0)	1 (10.0)	1 (5.3)
**Employment (*n*, %)**			
Employed	3 (33.3)	4 (40.0)	7 (36.8)
Unemployed	4 (44.4)	6 (60.0)	10 (52.6)
Retired	2 (22.2)	0 (0.0)	2 (10.5)
**HPN duration, y (median, IQR)**	10 (6, 35)	2.25 (1, 5)	5 (2.25, 9.5)
**HPN nights per wk**			
3 nights per wk	2 (22.2)	0 (0.0)	2 (10.5)
4 nights per wk	0 (0.0)	2 (20.0)	2 (10.5)
5 nights per wk	1 (11.1)	1 (10.0)	2 (10.5)
6 nights per wk	0 (0.0)	1 (10.0)	1 (5.3)
7 nights per wk	6 (66.6)	6 (60.0)	12 (63.2)
**Eat by mouth (*n*, %)**	8 (88.9)	4 (40.0)	12 (63.2)
**Diagnosis or indication for receiving HPN**^1^**(*n*, %)**			
Short bowel syndrome	6 (66.7)	3 (30.0)	9 (47.4)
Severe gastrointestinal dysmotility	1 (11.1)	7 (70.0)	8 (42.1)
Surgical complication	1 (11.1)	1 (10.0)	2 (10.5)
Intestinal failure	3 (33.3)	1 (10.0)	4 (21.1)
Other	3 (33.3)	2 (20.0)	5 (26.3)

HPN, home parenteral nutrition.

1 Patients may have multiple indications

Of the 11 clinicians who participated in the clinician focus groups, 8 (72.7%) were dietitians, 2 (18.2%) were nurse practitioners, and 1 (9.1%) was a physician. Clinicians cared for HPN patients for a median of 10 y (Q1, Q3 = 6, 13). Clinicians at the focus groups recognized that there is a lack of attention to sleep challenges experienced by patients due to time constraints attributed to the complexity of HPN management. Strategies to cope with sleep complaints such as nocturnal polyuria, noisy pump alarms, and line entanglement were often individualized. Common strategies included recommending HPN or intravenous hydration during the daytime, adjusting infusion schedules, rates, or volumes, whenever possible, using long, curly tubing to support mobility and to position the pump further away from the bedside, and replacing or troubleshooting pumps.

From the focus groups, an additional 5 recommendations were added based on lived experience, including 3 newly developed recommendations and 2 previously discarded recommendations.

### Knowledge test

Pre- and post-knowledge tests were completed by 10 additional community members. Demographic data of the knowledge test participants are presented in Table 1. Briefly, the average age of knowledge test participants was 39.8 (SD = 14.0), and 70% identified as female. Participants were supported by HPN for a median of 2.25 y (Q1, Q3 = 1, 5), and most participants (60%) ran their infusions 7 nights per week. The most common diagnoses for receiving HPN were severe gastrointestinal dysmotility (70%) followed by short bowel syndrome (30%). Two recommendations were deemed not useful by ≥40% of participants: “*A bedside commode can make bathroom trips quicker and easier*,” and “*If you smoke tobacco or vape, try quitting*” (Supplementary Table 2). Participants reported that bedside commodes were not feasible for everyone and opposed the idea of placing one in their rooms next to their medical equipment. The expert panel agreed to embed the recommendation within an existing one as it may be useful for some. Participants also reported that the recommendation pertaining to smoking was insensitive to the difficulty of smoking cessation and required rephrasing. Additional minor changes were made to another 24 items. There was a modest increase in scores from the knowledge test after presenting the healthy sleep practices [pre-test: 26.6 (SD = 1.2); post-test: 27.4 (SD = 1.5)]. In response to questions regarding the usefulness of the drafted sleep recommendations, most community members (90.0%) indicated that the recommendations were ‘very useful’ or ‘somewhat useful’ (Table 2). Additionally, all (100%) participants indicated that they would adopt the recommendations and share them with other members.

**TABLE 2:** Sleep health knowledge test results and self-reported usefulness of recommendations (*n*=10)

Knowledge test results	
**Pre-knowledge test score**^1^**(mean, SD)**	26.6 (1.2)
**Post-knowledge test score**^1^**(mean, SD)**	27.4 (1.5)
**Self-reported usefulness of recommendations**	
**Overall usefulness of recommendations (*n*, %)**	
Very useful	5 (50.0)
Somewhat useful	4 (40.0)
Somewhat unuseful	1 (10.0)
Very unuseful	0 (0.0)
**Likelihood of adopting recommendations (*n*, %)**	
Very likely	6 (60.0)
Somewhat likely	4 (40.0)
Somewhat unlikely	0 (0.0)
Very unlikely	0 (0.0)
**Likelihood of sharing recommendations with other HPN consumers(*n*, %)**	
Very likely	7 (70.0)
Somewhat likely	3 (30.0)
Somewhat unlikely	0 (0.0)
Very unlikely	0 (0.0)

HPN, home parenteral nutrition.

1 Test scores are presented as the number of correct responses out of 30 total.

The final evaluation by the expert panel included changes to the title, introductory materials, section sub headers, phrasing, and order of the recommendations. All members of the expert panel agreed with the final recommendations. The final resource included 36 healthy sleep practices spanning 4 categories: getting ready for bed, preparing the bedroom for sleep, daytime behaviors, and overall strategies for better sleep and a sleep diary (Figure 2, Supplementary Material 4, Supplementary Material 5).

## Discussion

To our knowledge, we curated the first set of healthy sleep practices tailored for consumers of HPN. This was facilitated by a multidisciplinary expert panel and a strategic collaboration with members of the HPN community and their clinicians. We anticipate that the wide distribution of these resource in partnership with patient advocacy and support groups, including the Oley Foundation, the largest nonprofit organization serving this community [27], will improve the sleep health of HPN consumers and increase attention to the central role of sleep on health and well-being.

Our mixed-methods, community-based participatory research study design was central to developing our resources as too few studies have been published on sleep in this population. By involving consumers of HPN and their clinicians in the design and data collection phases of the study, we were able to draw on lived experience from a heterogeneous patient population. Community representation was crucial considering the level of patient involvement in HPN management. A multidisciplinary panel with complementary expertise in nutrition support, sleep medicine, and community sleep health was also central to assessing new practices drawn from the lived experience and evaluating the relevance of general sleep guidelines for this population. Our integrative and iterative framework can be leveraged as a model for future community-based participatory research in nutrition support.

Our resource consists of 36 recommendations spanning 4 sections: getting ready for bed, preparing the bedroom for sleep, daytime behaviors, and overall strategies for better sleep. A total of 21 (58%) recommendations are largely consistent with general sleep hygiene recommendations [15]. The remaining 15 (42%) recommendations address specific sleep challenges experienced by HPN consumers including nocturnal polyuria, noise and light emitted from medical equipment, timing and consistency of infusion schedules, a cluttered bedroom, and medication interference. The expert panel agreed on the “softening” of language in recognition of the complexity of HPN management and indication by including statements such as “where possible,” particularly to recommendations that were more consistent with general sleep hygiene. In recognizing the heterogeneity in this population, “if you eat by mouth” was included as not all patients eat by mouth. The term “healthy sleep practices” was adopted based on consensus from another large sleep expert panel [17]. The resource's introductory statement encourages HPN consumers to implement an experimental approach to their sleeping habits by describing the guidelines as representative of the current evidence base, while suggesting a process of “trial and error.” We also demonstrated that the information on healthy sleep practices for HPN consumers was deemed useful by most participants and led to a modest increase in knowledge test scores before and after providing the healthy sleep practices.

### Implications and future directions

There currently is no guidance on sleep for HPN consumers and their clinicians. Thus, consumers of HPN are uniquely qualified for targeted sleep recommendations. Access to healthy sleep practices is considered a global public health priority [9,29]. Despite the wide recognition that overnight infusions of HPN elicit sleep disturbance, there have been few efforts aimed at mitigating this effect and no targeted guidance on healthy sleep practices [30]. Indeed, there is a common misconception that there are ample resources for patients receiving lifelong and life-sustaining HPN therapy [31]. There is evidence to suggest that patients eventually learn to self-cope with overnight infusions with time [32], but early guidance is imperative to not compromise the health and safety of patients upon initiating HPN [33]. Our study ultimately provides tools for consumers and their clinicians to support the sleep of their patients. It was estimated in 2017 that >25,000 patients in the United States are dependent on HPN [34]. The success of our study is contingent on wide dissemination of these recommendations.

We recommend that clinicians help patients anticipate sleep issues and routinely inquire about sleep. Clinicians must be aware of common disruptors of sleep, such as alarm systems of programmable pumps [33], and options to mitigate these issues as outlined in the handout, for example replacing pole-mounted pumps with portable pumps [35]. Factors, such as high infusion rate, large volume, and some formula composition may also contribute to sleep complaints and may need to be re-evaluated if sleep problems persist.

We did not assess whether the recommendations developed for this project change behavior or improve sleep. It is important to note that although general sleep hygiene recommendations have been proven to be effective in improving sleep, some recommendations included in this specific project are based on anecdotal evidence and thus should not be considered evidence-based. However, sleep education is associated with improved sleep outcomes in other populations (e.g., hospitalized patients, elderly adults, college students, and children) [[36], [37], [38], [39]]. Future research should examine the impact of the recommendations on sleep outcomes, quality of life, and clinical endpoints.

### Limitations

Important limitations need to be considered. Our recruitment through social media and patient advocacy and support groups was susceptible to volunteer bias. Indeed, the majority of our study participants were women and White. It is possible that we may have captured HPN consumers primarily of higher socioeconomic status with online access who are able to partake in such research. Therefore, our sample of study participants may not be representative of the broader HPN community due to our approach to recruitment, our inclusion/exclusion criteria, or our study approach. It is worth noting that community members partaking in our focus groups were also older and had been infusing HPN for longer than those partaking in the sleep knowledge test, which may influence our findings. Due to this and our generally small sample size, all findings presented in this study should be considered preliminary and may not be generalizable to all consumers. Similar to recent qualitative studies relying on online recruitment, our study was prone to imposter participants (i.e., fake participants) [24]. We considered HPN in its broad context, without differentiating the diversity of HPN indication or composition including fluid, dextrose, lipid, or amino acids. Our study focused on a single, yet vulnerable patient population; however, some aspects of our handouts may be generalized to other populations receiving overnight enteral nutrition (tube feeds), intravenous hydration therapy, home chemotherapy, and dialysis [40]. Our resources were translated into Spanish, but translations into other languages are required to increase accessibility.

## Conclusion

In summary, we developed tailored sleep resources targeting a vulnerable population to promote the long-term well-being of HPN consumers. We anticipate that democratizing sleep knowledge through our partnership with patient advocacy and support groups will improve the safety, mood, and overall health of consumers of HPN.

## Author contributions

The authors’ responsibilities were as follows – AR: Methodology, Formal analysis, Investigation, Data Curation, Writing – original draft preparation, Visualization, Project administration. CS: Conceptualization, Methodology, Writing – Review & Editing. BJ: Conceptualization, Investigation, Writing – Review & Editing. SM: Conceptualization, Investigation, Writing - Review & Editing. John Winkelman: Writing - review & editing. CC: Conceptualization, Methodology, Writing - Review & Editing. MW: Conceptualization, Methodology, Writing - Review & Editing. HSD: Conceptualization, Methodology, Formal analysis, Investigation, Data Curation, Writing - original draft preparation, Visualization, Supervision, Funding acquisition. All authors have read and approved the final manuscript. We thank Chloe Liu and Sierra Chichester for their useful suggestions and contributions to the project and the construction of the manuscript.

## Conflict of Interest

MFW is a scientific advisor for Takeda Pharmaceuticals (Lexington, MA) and VectivBio (Basel, Switzerland). All other authors report no conflicts of interest.

## Funding

This work was supported by the American Academy of Sleep Medicine (AASM) Foundation [grant 293-CS-22] awarded to HSD. This work was also supported by the National Institute of Health [grant number R00HL153795 to HSD]. The funding sources had no involvement in study design.

## Data availability

Data described in the manuscript, code book, and analytic code will be made available to researchers upon request pending approval for access to confidential data as determined by the Mass General Brigham Human Research Office/Institutional Review Board at Mass General Brigham.

## References

[1] Luyster F.S., Strollo P.J., Zee P.C., Walsh J.K. (2012). Boards of Directors of the American Academy of Sleep Medicine and the Sleep Research Society, Sleep: a health imperative. Sleep.

[2] Baranwal N., Yu P.K., Siegel N.S. (2023). Sleep physiology, pathophysiology, and sleep hygiene. Prog. Cardiovasc. Dis..

[3] Medic G., Wille M., Hemels M.E. (2017). Short- and long-term health consequences of sleep disruption. Nat. Sci. Sleep..

[4] Van Dongen H.P.A., Maislin G., Mullington J.M., Dinges D.F. (2003). The cumulative cost of additional wakefulness: dose-response effects on neurobehavioral functions and sleep physiology from chronic sleep restriction and total sleep deprivation. Sleep.

[5] Ford E.S., Wheaton A.G., Chapman D.P., Li C., Perry G.S., Croft J.B. (2014). Associations between self-reported sleep duration and sleeping disorder with concentrations of fasting and 2-h glucose, insulin, and glycosylated hemoglobin among adults without diagnosed diabetes. J. Diabetes.

[6] Cappuccio F.P., Cooper D., D’Elia L., Strazzullo P., Miller M.A. (2011). Sleep duration predicts cardiovascular outcomes: a systematic review and meta-analysis of prospective studies. Eur. Heart. J..

[7] Prather A.A., Marsland A.L., Hall M., Neumann S.A., Muldoon M.F., Manuck S.B. (2009). Normative variation in self-reported sleep quality and sleep debt is associated with stimulated pro-inflammatory cytokine production. Biol. Psychol..

[8] Sheehan C.M., Frochen S.E., Walsemann K.M., Ailshire J.A. (2019). Are U.S. adults reporting less sleep?: Findings from sleep duration trends in the National Health Interview Survey, 2004-2017. Sleep.

[9] Lim D.C., Najafi A., Afifi L., Bassetti C.L.A., Buysse D.J., Han F. (2023). The need to promote sleep health in public health agendas across the globe. Lancet. Public. Health..

[10] Espie C.A. (2022). The “5 principles” of good sleep health. J. Sleep. Res..

[11] Albakri U., Drotos E., Meertens R. (2021). Sleep health promotion interventions and their effectiveness: an umbrella review. Int. J. Environ. Res. Public. Health..

[12] Irish L.A., Kline C.E., Gunn H.E., Buysse D.J., Hall M.H. (2015). The role of sleep hygiene in promoting public health: a review of empirical evidence, Sleep. Med. Rev..

[13] Mastin D.F., Bryson J., Corwyn R. (2006). Assessment of sleep hygiene using the Sleep Hygiene Index. J. Behav. Med..

[14] Wheaton A.G., Jones S.E., Cooper A.C., Croft J.B. (2018). Short sleep duration among middle school and high school students - United States, 2015. Morb. Mortal. Wkly. Rep..

[15] Edinger J.D., Arnedt J.T., Bertisch S.M., Carney C.E., Harrington J.J., Lichstein K.L. (2021). Behavioral and psychological treatments for chronic insomnia disorder in adults: an American Academy of Sleep Medicine systematic review, meta-analysis, and GRADE assessment. J. Clin. Sleep. Med..

[16] Jung S.Y., Kim H.S., Min J.Y., Hwang K.J., Kim S.W. (2019). Sleep hygiene-related conditions in patients with mild to moderate obstructive sleep apnea. Auris. Nasus. Larynx..

[17] Shriane A.E., Rigney G., Ferguson S.A., Bin Y.S., Vincent G.E. (2023). Healthy sleep practices for shift workers: consensus sleep hygiene guidelines using a Delphi methodology. Sleep.

[18] Kumpf V.J. (2019). Challenges and obstacles of long-term home parenteral nutrition. Nutr. Clin. Pract..

[19] Bering J., DiBaise J.K. (2022). Home parenteral and enteral nutrition. Nutrients.

[20] Dashti H.S., Rhyner J.J., Mogensen K.M., Godbole M., Saxena R., Compher C. (2023). Infusion timing and sleep habits of adults receiving home parenteral and enteral nutrition: a patient-oriented survey study. JPEN J. Parenter. Enteral. Nutr..

[21] Dashti H.S., Godbole M., Chen A., Mogensen K.M., Leong A., Burns D.L. (2022). Sleep patterns of patients receiving home parenteral nutrition: a home-based observational study. JPEN J. Parenter. Enteral. Nutr..

[22] Huisman-de Waal G., Bazelmans E., van Achterberg T., Jansen J., Sauerwein H., Wanten G. (2011). Predicting fatigue in patients using home parenteral nutrition: a longitudinal study. Int. J. Behav. Med..

[23] Schönenberger K.A., Reber E., Huwiler V.V., Dürig C., Muri R., Leuenberger M. (2023). Quality of life in the management of home parenteral nutrition. Ann. Nutr. Metab..

[24] Ridge D., Bullock L., Causer H., Fisher T., Hider S., Kingstone T. (2023). “Imposter participants” in online qualitative research, a new and increasing threat to data integrity? Health. Expect.

[25] Tong A., Sainsbury P., Craig J. (2007). Consolidated criteria for reporting qualitative research (COREQ): a 32-item checklist for interviews and focus groups. Int. J. Qual. Health. Care..

[26] Wong L.P. (2008). Focus group discussion: a tool for health and medical research, Singapore. Med. J..

[27] Andolina J.M., Metzger L.C., Bishop J. (2019 Dec). The Oley Foundation and consumer support groups. Gastroenterol. Clin. North Am..

[28] Chopy K., Winkler M., Schwartz-Barcott D., Melanson K., Greene G. (2015). A qualitative study of the perceived value of membership in the Oley Foundation by home parenteral and enteral nutrition consumers. J. Parenter. Enteral. Nutr..

[29] Buysse D.J. (2014). Sleep health: can we define it? Does it matter?. Sleep.

[30] Huisman-de Waal G., Schoonhoven L., Jansen J., Wanten G., van Achterberg T. (2007). The impact of home parenteral nutrition on daily life-a review. Clin. Nutr..

[31] Lowthian T.J. (2023). Why are we settling? Improving home parenteral nutrition quality of life outcomes. J. Parenter. Enteral. Nutr..

[32] Girke J., Seipt C., Markowski A., Luettig B., Schettler A., Momma M. (2016). Quality of life and nutrition condition of patients improve under home parenteral nutrition: an exploratory study. Nutr. Clin. Pract..

[33] Lehoux P. (2004). Patients’ perspectives on high-tech home care: a qualitative inquiry into the user-friendliness of four technologies, B.M.C. Health. Serv. Res..

[34] Mundi M.S., Pattinson A., McMahon M.T., Davidson J., Hurt R.T. (2017). Prevalence of home parenteral and enteral nutrition in the United States. Nutr. Clin. Pract..

[35] Saqui O., Fernandes G., Allard J.P. (2014). Quality of life analysis during transition from stationary to portable infusion pump in home parenteral nutrition patients: a Canadian experience. Nutr. Clin. Pract..

[36] Herscher M., Mikhaylov D., Barazani S., Sastow D., Yeo I., Dunn A.S. (2021). A sleep hygiene intervention to improve sleep quality for hospitalized patients. Jt. Comm. J. Qual. Patient. Saf..

[37] Tucker R.M., Contreras D.A., Carlson B.R., Carter A., Drake C.L. (2021). Sleep Education for Elders Program (SLEEP): promising pilot results of a virtual, health educator-led, community-delivered sleep behavior change intervention. Nat. Sci. Sleep..

[38] Hershner S., O’Brien L.M. (2018). The impact of a randomized sleep education intervention for college students, J. Clin. Sleep. Med.

[39] Gruber R., Somerville G., Bergmame L., Fontil L., Paquin S. (2016). School-based sleep education program improves sleep and academic performance of school-age children, Sleep. Med..

[40] Nocturnal Home Hemodialysis. Home Dialysis Central n.d. https://homedialysis.org/home-dialysis-basics/nocturnal-home-hemodialysis (accessed October 9, 2023).

