# Supplementary material for: Healthy Sleep Practices for Consumers of Home Total Parenteral Nutrition: A Mixed-Methods Community-Based Participatory Study
Source: Curr Dev Nutr. 2024 Apr 5;8(5):102155. doi: 10.1016/j.cdnut.2024.102155 (PMC11059546; doi:10.1016/j.cdnut.2024.102155)
Supplement: Multimedia component 1 [file mmc1.pdf]

Supplementary Material

Supplementary Material 1. Patient Focus Group Guide

Supplementary Material 2. Clinician Focus Group Guide

Supplementary Material 3. Sleep Health Knowledge Test

Supplementary Material 4. Healthy Sleep Practices for Consumers of Home TPN

Supplementary Material 5. Sleep Diary for Consumers of Home TPN

Supplementary Material 6. Healthy sleep practices for consumers of home TPN (Spanish translation)

Supplementary Material 7. Sleep Diary for Consumers of Home TPN (Spanish translation)

Supplementary Table 1. Synthesis of Sleep Health Recommendations for Consumers of Home Parenteral Nutrition

Supplementary Table 2. Usefulness of 37 Sleep Recommendations Tailored for Home Parenteral Nutrition Consumers

Supplementary Material 1. Community Member Focus Group Guide

Community Member Focus Group Guide

1. **Introduction**

Consent forms for focus group participants are completed in advance by all those seeking to participate. Below is a summary of the information in the consent form that focus group organizers and facilitators should use to make sure participants understand the information in the consent form.

- **Welcome**
  - *Welcome everyone and thank you for agreeing to participate. We are very interested to hear your valuable experience with sleep while receiving overnight infusions of total parenteral nutrition (TPN).*
- **Purpose, why you were selected**
  - *The purpose of this study is to learn how you have coped with sleeping while receiving home TPN. You are here because you have agreed to share your experience with your infusions. Your experiences are valuable to us and we want to hear and learn from you. We also hope to learn more about the support that you may have received or sought to get better sleep. This information will help us develop some handouts for the community on best sleep practices.*
- **Agenda, consent, remuneration**
  - *This focus group should take 60 minutes. We will start with some ground rules and expectations, an explanation of the process, we'll share introductions, and then our discussion questions.*
  - *At the end of the discussion, you will each receive a $100 check mailed to your home address for your participation.*
- **Consent, recording**
  - *We would like to remind you that the information and stories you are sharing with us are completely confidential, and we will not associate your name with anything you share in the focus group.*
  - *We would like to record the focus groups so that we can make sure to capture the thoughts, opinions, and ideas we hear from the group. No names will be attached to the focus groups and the recordings will be destroyed as soon as they are transcribed.*
  - *You may refuse to answer any question or withdraw from the study at any time.*
  - *We understand how important it is that this information is kept private and confidential. We will ask participants to respect each other's confidentiality and responses.*
  - *If you have any questions now or after, you can always contact a study team member like myself, whose names and phone numbers are on the fact sheet we have shared.*

2. **Explanation of the process**

- Ask the group if anyone has participated in a focus group before. Explain that focus groups are being used in health and human services research.
  - *Has anyone here ever participated in a focus group before?*
    - *If no: Focus groups are often used in health and human services research*

to gain insight from specific populations on a topic.

- About focus groups
    - We learn from you (positive and negative)
        - *We want to learn from you and hear your individual experiences since you are the experts.*
    - Not trying to achieve consensus, we're gathering information
        - *We understand that everyone here will have different experiences and viewpoints. We do not expect you all to agree and we are not looking to achieve consensus. Rather, this discussion will help us gather information.*
    - No virtue in long lists: we're looking for priorities
        - *We specifically want to learn what your most pressing concerns are to help us with the project.*
- Logistics and ground rules
    - Focus group will last about 60 minutes.
    - Everyone is expected to participate.
        - *We want to hear from all of you, so we will moderate the discussion to ensure all voices are heard. We may call on individuals who have not had a chance to speak or who we have not heard much from.*
    - To ensure everyone has a chance to contribute, we will call out on people. If you have something to add, please raise your hand using the hand button.
    - Information provided in the focus group will be kept confidential.
    - Stay with the group, please reduce distractions, and silence or turn off cell phones if possible.
        - *One person will speak at a time. Please respect everyone's turn to speak.*
    - Staying on track.
        - *If the discussion is veering off track, we may intervene and redirect back to the topic in the interest of time.*
    - *Does anyone have any questions before we start the recording?*

3. **Start the recording**
4. **Ask the group if there are any questions before we get started, and address those questions.**
5. **Introductions, icebreaker**
    - Go around: 1) first name only, and 2) how long you have been on home TPN.
6. **Questions**

Discussion begins, make sure to give people time to think before answering the questions and don't move too quickly. Use the probes to make sure that all issues are addressed but move on when you feel you are starting to hear repetitive information.

General

1. Let's start the discussion by talking about the timing of your TPN infusions.
    a. *Were you involved in setting the times for your overnight infusions?*
        i. What was that like for you?
        ii. What would you have liked to have done?
    b. Advantages to the current timing of your infusions?
    c. Disadvantages or challenges related to the current timing of your infusions?

Sleep at start of infusion – *transition into talking about sleep at the start of your infusions*

2. Can you please tell us about the current quality of your sleep?
3. Tell me about your sleep since you started home TPN.
    a. Has your sleep quality changed as a result of home TPN (particularly overnight)?
        i. If so, in what way?
        ii. How would you describe your sleep during your infusions?
        iii. Has your sleep improved? – if yes, probe with the following:
            1. *In what way? Please describe what you mean.*
            2. *How long did it take it to improve?*
            3. *What helped your sleep improve? (ex. sleep strategies, something else…)*
        iv. Has your sleep worsened? – if yes, probe with the following:
            1. *Can you please share more?*
            2. *Why do you think your sleep has not improved?*
        v. If not, please tell me more.
    b. What would be useful for you to support the sleep health of future home TPN consumers?

Summarize what participants have shared so far about their sleep.

4. What sort of questions did you have about your sleep particularly upon starting home TPN?
    a. *Please give me an example.*
    b. *When did these questions come about? (before overnight infusions, during..)*
    c. *Where did you go to get these questions answered?*
    d. *Were these sources of information helpful in getting your questions answered?*
5. Upon starting home TPN, were you told about your sleep?
    a. *If yes,*
        i. *What was discussed with you?*
        ii. *What are some things you were told about sleep when you first started your infusions?*
        iii. *Please give me an example. Describe what you mean.*
    b. If no,
        i. Please describe what that was like for you.
        ii. How did that make you feel?
        iii. What would you have liked to have heard?
6. Has the sleep of others living in your household been impacted by your overnight infusions?
    a. *If yes, how?*
        i. How have they coped with the infusions?
    b. How can members of your household be supportive of your healthy sleep habits?

<u>Sleep strategies</u> – *transition into talking about coping strategies for improved sleep.*

7. Have you tried any strategies to help you sleep better?
    a. If so,
        i. *What strategies have you tried?*
        ii. How have these strategies helped you?
        iii. What has made these strategies helpful for you?
        iv. Where did you learn about these strategies?
            1. *Who did you reach out to or contact to get that information?*
    b. If not,

        i. *Can you please share why?*
        ii. *What would make you more likely to adopt sleep strategies?*

8. Are there any strategies that have **not** worked for you?
    a. *Please give me an example.*
    b. *Why or why not?*

Summarize strategies at this point and obtain participant feedback on your understanding.

Recommendation specific questions – We will now transition to asking about some of the recommendations we are considering (as found practical by our research team).

9. Would you say that your infusions affect your sleep schedule?
    a. How is the timing of your sleep determined (in relation to your infusions)?
    b. *For some people, infusions can have a positive impact on sleep by helping regulate their sleep schedule. Has that happened for anyone? In what way?*
    c. Have you tried to change the hours you infuse your TPN to improve sleep?
        i. *Please describe the changes you made.*
        ii. *Were these changes self-motivated or did you seek guidance from a clinician?*
        iii. *Has this change helped?*
    d. How practical do you think it would be for you to sleep at the same time every night, even if not infusing?
10. How do you prepare yourself for bed?
    a. Do you taper your infusions before bedtime?
        i. If so,
            1. How does this affect your bedtime routine?
            2. How does this affect time spent in bed?
                a. *Does this encourage you to spend more time in bed?*
            3. Please describe your bedtime routine.
                a. *How do you spend your time in bed before falling asleep?*
    b. Do you find your bedtime routine helpful in preparing you for sleep?
        i. *How so?*
        ii. *Why or why not?*
    c. What activities do you think should be included in a bedtime routine (as a TPN consumer)?
11. How would you describe your sleeping environment when you are infusing?
    a. How feasible is it for you to sleep in a dark, quiet, room?
        i. Do you find light-at-night exposure disruptive to your sleep?
            1. If so,
                a. *How do you address light-at-night exposure?*
            2. If not,
                a. *Why is that? (ex. Prioritize safety, comfort?)*
    b. Does your infusion pump influence how you sleep in any way?
        i. Please explain.
        ii. If disturbing to sleep,
            1. *What types of strategies help you sleep with your infusion pump?*
            2. *How do you reduce or mask the noise? (ex. Sleep aids, noise reduction machines)*

Summarize and obtain participant feedback on your understanding.

12. How often/how many times do you wake up at night to use the bathroom?
    a. What causes you to wake up at night to use the bathroom?
        i. *For example, do you wake up to urinate, to empty your ostomy/because of diarrhea, both, other reasons?*
    b. Has being on TPN affected this pattern (the number of times you wake up at night to use the bathroom)?
        i. Please explain.
    c. How has waking up at night to use the bathroom affected your overall sleep quality?
        i. *Have you tried any strategies to overcome this?*
        ii. *Some possible strategies include decreasing barriers to the toilet (ex. Sleeping closest to the restroom, light source) or devices like commodes, catheter bottles, and toilet chairs. Would you describe these as practical solutions?*
13. Are you aware of any strategies or therapies that promote sleep onset or maintenance? *(ex. Stress management, mindfulness/meditation, music therapy, CBT)*
    a. Have you tried any of these strategies?
        i. *How have they worked for you?*
        ii. *How have they not worked for you?*
        iii. *Please describe their impact on your sleep quality.*
    b. Would you describe these as feasible solutions?
        i. *Why or why not?*

Summarize: describe the nature of these strategies (behavioral, cognitive, pharmacological)

14. How does your central line affect how you sleep, if at all?
    a. How has your central line affected your sleep quality?
    b. Did you have any concerns about your central line upon starting home TPN?
        i. *For example - malpositioning, tubing becoming tangled when you sleep, tubing limiting sleep positioning/comfort?*
        ii. *Tell me more.*
15. Do you nap during the day at all?
    a. If so,
        i. How often do you nap?
        ii. What causes you to nap during the day? (esp if frequent)
            1. *Is this a result of inadequate sleep at night or other reasons?*
            2. *How does your sleep at night affect daytime napping?*
                a. Does napping help support your sleep health, overall?
            3. *Why or why not?*
        iii. Has being on TPN changed your daytime napping habits?
            1. If so,
                a. *Please tell me more.*
            2. If not,
                a. *Why is that?*

Summarize and obtain participant feedback on your understanding.

<u>Advice, Suggestions</u>

16. What would you have liked to know about sleep upon first starting TPN at home?

17. What advice do you have for people just starting overnight infusions?
    a. *Would you say you learned this from your own experience or from external resources?*
18. If you have questions about your sleep, who did you go to in order to have these questions answered?
    a. *Which resources or individuals have you found most helpful regarding your sleep?*
19. How can clinicians help support your sleep health?

Summarize impressions at this point and obtain participant feedback.

<u>Anything else?</u>

20. What else would you like to share that we didn't discuss that you think would be important for us to know?
21. Is there anything else you would like to know about sleep?
22. Do you have any additional comments?
23. Are there any other considerations you believe we should make when developing sleep resources?
    a. *Ex. Feasibility, accessibility, cultural factors, comorbidities (anxiety, depression, eating disorders..)*

7. End

24. Summarize overall impressions one last time, make sure you have an accurate understanding of what was said.
25. Thank the participants, remind of remuneration, and end the recording.

Supplementary Material 2. Clinician Focus Group Guide

Clinician Focus Group Guide

1. Introduction

Consent forms for focus group participants are completed in advance by all those seeking to participate. Below is a summary of the information in the consent form that focus group organizers and facilitators should use to make sure participants understand the information in the consent form.

- **Welcome**
  - *Welcome everyone and thank you for agreeing to participate. We are very interested to hear your valuable experience with addressing the sleep health of patients receiving overnight infusions of total parenteral nutrition (TPN).*
- **Purpose, why you were selected**
  - *The purpose of this study is to learn how you have guided patients with sleeping while receiving at home TPN, if at all. We know that not all providers do provide information on sleep, which is okay. We are just trying to understand why to best support providers and patients. You are here because you have agreed to share your experience treating patients receiving infusions. Your experiences are valuable to us and we want to hear and learn from you. This information will help us develop some handouts for the community on best sleep practices.*
- **Agenda, consent, remuneration**
  - *This focus group should take 60 minutes. We will start with some ground rules and expectations, an explanation of the process, we'll share introductions, and then our discussion questions.*
  - *At the end of the discussion, you will each receive a $50 gift card for your participation.*
- **Consent, recording**
  - *We would like to remind you that the information and stories you are sharing with us are completely confidential, and we will not associate your name with anything you say in the focus group.*
  - *We would like to record the focus groups so that we can make sure to capture the thoughts, opinions, and ideas we hear from the group. No names will be attached to the focus groups and the recordings will be destroyed as soon as they are transcribed.*
  - *You may refuse to answer any question or withdraw from the study at any time.*
  - *We understand how important it is that this information is kept private and confidential. We will ask participants to respect each other's confidentiality and responses.*
  - *If you have any questions now or after, you can always contact a study team member like myself, whose names and phone numbers are on the fact sheet we have shared.*

2. Explanation of the process

- Ask the group if anyone has participated in a focus group before. Explain that focus groups are being used in health and human services research.
  - *Has anyone here ever participated in a focus group before?*
    - *If no: Focus groups are often used in health and human services research*

to gain insight from specific populations on a topic.

- About focus groups
    - We learn from you (positive and negative)
        - *We want to learn from you and hear your individual experiences since you are the experts.*
    - Not trying to achieve consensus, we're gathering information
        - *We understand that everyone here will have different experiences and viewpoints. We do not expect you all to agree and we are not looking to achieve consensus. Rather, this discussion will help us gather information.*
    - No virtue in long lists: we're looking for priorities
        - *We specifically want to learn what the most pressing concerns of your patients are and what is most relevant to you as clinicians guiding TPN consumers.*
- Logistics and ground rules
    - Focus group will last about 60 minutes.
    - Everyone is expected to participate.
        - We *want to hear from all of you, so we will moderate the discussion to ensure all voices are heard. We may call on individuals who have not had a chance to speak or who we have not heard much from.*
    - To ensure everyone has a chance to contribute, we will call out on people. If you have something to add, please raise your hand using the hand button.
    - Information provided in the focus group will be kept confidential.
    - Stay with the group, please reduce distractions, and silence or turn off cell phones if possible.
        - *One person will speak at a time. Please respect everyone's turn to speak.*
    - Staying on track.
        - *If the discussion is veering off track, we may intervene and redirect back to the topic in the interest of time.*
    - *Does anyone have any questions before we start the recording?*

3. **Start the recording**
4. **Ask the group if there are any questions before we get started, and address those questions.**
5. **Introductions, icebreaker**
    - Go around: 1) first name only, and 2) what is your role as a clinician (*ex. NP/RN, MD, PA-C, RD/RDN*)?
6. **Questions**

Discussion begins, make sure to give people time to think before answering the questions and don't move too quickly. Use the probes to make sure that all issues are addressed but move on when you feel you are starting to hear repetitive information.

<u>General, sleep health knowledge</u>

1. Let's start the discussion by talking about your general knowledge on sleep health, if any.
    a. *What does the term sleep health mean to you?*
    b. *How would you describe your understanding of sleep health?*
    c. *Where have you gained most of your knowledge on sleep health?*
2. How often do sleep issues come up with your patients? Or tell me about how often sleep comes up with your patients.

3. Please describe conversations about sleep that you have had with your patients, if any.
    a. Who raises these concerns?
        i. *For example - do you routinely inquire about sleep issues or screen for sleep disorders (ex. Insomnia), or do patients bring them up themselves?*
    b. What is the role of infusions in sleep concerns for patients?
        i. *Or what are the sleep problems related to?*
            1. For example – GI causes, diarrhea, ostomy leakage, overnight TPN infusions.
        ii. Do these tend to be preexisting sleep problems, sleep issues resulting from overnight infusions, or other reasons?
    c. If no conversations:
        i. *Can you please explain why sleep is not routinely discussed with your patients? (We understand that it can be very difficult to talk to patients about everything in the interest of time, among other reasons – patients don't bring it up, not a priority, not within clinician's scope of practice/expertise/responsibility?).*
4. What sort of questions do patients have about their sleep?
    a. *How do you address these sleep concerns? (ex. refer to someone else, redirect patient to resources..)*
5. What are some things you shared with your patients about their sleep upon them first starting their overnight infusions?
    a. *Please give me an example.*
    b. If nothing/very little:
        i. And why is that?

Summarize what has been shared so far.

<u>Strategies</u>

6. How have you guided patients with sleeping while receiving home TPN, if at all?
7. What strategies have you shared with your patients in order to help them achieve better sleep?
    a. *Where did you learn about these strategies?*
    b. *Were these strategies helpful to your patients?*
        i. How do you know?
        ii. Why do you think that is?
        iii. Please give me an example.
8. What strategies have you shared with your patients that have **not** been helpful to them?
    a. *How do you know?*
    b. *Why do you think that is?*
    c. *Please give me an example.*

Summarize strategies at this point and obtain participant feedback on your understanding.

<u>Recommendation specific questions</u> - We will now transition to asking about some of the recommendations we are considering (as found practical by our research team).

9. How do you determine the timing of your patients' infusions?
    a. *Would you consider prolonging (or recommend prolonging) the timing for larger volumes to reduce nocturia?*

10. How do you address the fragmented, interrupted sleep of your patients?
    a. *How do you guide patients around sleep duration (7-9 hours) considering wake after sleep onset challenges?*
11. How do you address the challenge of nocturnal polyuria amongst your patients?
    a. What types of coping strategies do you recommend?
    b. *Please describe the feasibility of oral rehydration therapy throughout the day to reduce fluid intake before bedtime.*
    c. *Do you recommend pharmacological strategies to facilitate fluid absorption, reduce overall IV fluid dependency amongst your patients, or discontinue TPN?*
        i. *Please describe.*
        ii. *Tell me more.*
12. How do you guide new patients around managing or adapting to their infusion pumps (*regarding the light and noise*)?
    a. *What types of resources or strategies do you suggest?*
    b. *Do you think your patients find these strategies helpful?*
        i. *Why or why not?*
13. Daytime napping is common amongst home TPN patients in response to inadequate sleep at night. How do you guide patients around napping?
    a. *What would you recommend to patients for taking an effective nap?*

Summarize impressions at this point and obtain participant feedback.

14. How familiar are you with behavioral therapies to promote sleep onset or maintenance, such as stress management, mindfulness/meditation, music therapy, or CBT?
    a. If familiar,
        i. How often do you recommend such behavioral therapies?
        ii. Would you describe these as feasible solutions?
            1. *Why or why not?*
15. How do you guide new home TPN patients around managing their central line?
    a. How do you minimize patients' concerns about or fears of central line malpositioning and tube tangling?
16. How would you describe the role of exercise in supporting sleep health among your patients?
    a. *What types of activities might you recommend?*

Summarize impressions at this point and obtain participant feedback.

<u>Advice, suggestions</u>

17. What would you have liked to know about sleep to help you take better care of patients receiving overnight infusions of TPN?
    a. *How do you think we can support other clinicians to support the sleep health of patients receiving overnight infusions of TPN?*
18. If patients have questions about their sleep, who do you refer them to in order to have these questions answered?
    a. *Which resources have your patients found most helpful to have their sleep questions answered?*
    b. *Please give me an example.*

Summarize impressions at this point and obtain participant feedback.

<u>Anything else?</u>

19. What else would you like to share that we didn't discuss that you think would be important for us to know?
20. Is there anything else you would like to know about sleep?
21. Do you have any additional comments?
22. Are there any other considerations you believe we should make when developing sleep resources?
    a. *Ex. Feasibility, accessibility, cultural factors, comorbidities (anxiety, depression, eating disorders..)*

7. End

23. Summarize overall impressions one last time, make sure you have an accurate understanding of what was said.
24. Thank the participants and end the recording.

Supplementary Material 3. Sleep Health Knowledge Test

Sleep Health Knowledge Test

Please answer the following 30 questions to test your knowledge of sleep. This test should take up to 15 minutes to complete. Answer each question to the best of your ability.

Please read each of the following statements and mark whether you think it is true or false:

	**True**	**False**
**1. All adults need 8 hours of uninterrupted sleep.**	True	False
**2. Having consistent sleep and wake times helps to promote good sleep.**	True	False
**3. A warm room (70 degrees Fahrenheit) is recommended for better sleep.**	True	False
**4. Blue light activates melatonin, the hormone that helps you fall asleep.**	True	False
**5. The nicotine in tobacco products such as cigarettes helps you stay asleep.**	True	False
**6. Alcohol before bed helps with a good night's sleep.**	True	False
**7. Staying physically active can promote good sleep.**	True	False
**8. If you eat by mouth, a light snack before bed disturbs your sleep.**	True	False
**9. Getting outside during the day is helpful for your sleep.**	True	False
**10. Relaxation techniques can help clear the mind and promote sleep.**	True	False

**11. Unwinding 4 hours before bedtime is recommended.**	True	False
**12. Sleep does not affect your mood or ability to remember things the next day.**	True	False
**13. Going to bed before you are sleepy is often recommended.**	True	False
**14. It is best to sleep in a dark and quiet room.**	True	False
**15. Over the counter melatonin pills are generally safe to use occasionally as a sleep aid.**	True	False
**16. Tracking your sleep may help improve sleep patterns overall.**	True	False
**17. Taking a 2-hour daytime nap before 3pm will not disrupt your nighttime sleep.**	True	False
**18. If you eat by mouth, later mealtimes are recommended for better sleep.**	True	False
**19. If you eat by mouth, consuming caffeine too close to bedtime is not recommended.**	True	False
**20. It is best to stay in bed, even when it is difficult to fall asleep.**	True	False
**21. Certain medications can affect sleep and should be reviewed with healthcare providers for sleep interference.**	True	False
**22. Cognitive Behavioral Therapy for Insomnia (CBT-I) is not an effective treatment for insomnia and related sleep problems.**	True	False

**23. A relaxing bedtime routine may include checking your email and watching the news.**	True	False
**24. Over the counter sleep medications help with sleep only after long term use.**	True	False
**25. White noise machines are an example of a sleep aid that may help in getting to or maintaining sleep.**	True	False
**26. Stressful situations should be avoided before bed.**	True	False
**27. Sleep hygiene includes brushing your teeth before bed.**	True	False
**28. Spending a lot of time in bed during the day has no effect on nighttime sleep.**	True	False
**29. It is best practice to use the bathroom before going to bed.**	True	False
**30. Healthy sleep habits are universal and look the same for everyone.**	True	False

Knowledge Test Answer Key:

1, F; 2, T; 3, F; 4, F; 5, F; 6, F; 7, T; 8, F; 9, T; 10, T; 11, F; 12, F, 13, F; 14, T; 15, T; 16, T; 17, F; 18, F; 19, T; 20, F; 21, T; 22, F; 23, F; 24, F; 25, T; 26 T; 27, T; 28, F; 29, T; 30, F

Supplementary Material 4. Healthy sleep practices for consumers of home TPN

Getting better sleep when using TPN at home

Getting enough quality sleep is important for physical health and mental well-being. However, sleep disturbance is common for consumers of TPN at home, especially when TPN infusions happen at night during sleep.

The healthy sleep practices listed below may help you sleep better. The following guidance is based on scientific evidence and input from community members, including TPN consumers and healthcare providers. Improving sleep takes time. Pick a couple of changes that you can make based on the list below.

If your sleep problems persist, please talk to your healthcare team or a sleep specialist about other options.

Getting ready for bed

The things you do before going to bed can help prepare your body to sleep.

1. **Aim for 7 to 9 hours of sleep.** A total of 7 to 9 hours of sleep is the recommended duration for most adults; however, you may need more or fewer hours of sleep. Keep in mind that this time reflects the time you spend asleep, and not the time you spend in bed. You may need to spend more time in bed to get 7 to 9 hours of sleep. This duration also does not need to be continuous or uninterrupted. It is not unusual to wake up several times in the middle of the night with TPN. If you are struggling with waking up too frequently or falling back asleep in the middle of the night, talk to your healthcare team or a sleep specialist.
2. **Go to sleep and wake up at around the same time each day, even on weekends, days when you are off work, and on nights when you may not be running your TPN.** A regular sleep schedule helps regulate your biological clock by signaling to your body when it is time to sleep. Having consistent sleep and wake times helps you fall asleep and wake up when you want to.
3. **Maintain a TPN infusion schedule that is as consistent as possible by starting your TPN at the same time on days when you run your TPN.** Consistent TPN infusion times may also help prepare your body for better sleep. However, it might be stressful to have a fixed TPN schedule. Discuss the possibility of a flexible TPN schedule with your healthcare team.
4. **Do not go to bed unless you are ready to fall asleep.** It is best to wait until you are ready to fall asleep before going to bed. This will help you avoid lying awake in bed for too long, which may be frustrating. If you think that you may be struggling with insomnia, talk to your healthcare team or a sleep specialist.
5. **Avoid spending too much time in bed.** Use your bed only for sleep and intimacy. There may be exceptions, for example, if you are feeling unwell or

recovering from surgery.

6. **If you are unable to fall asleep after 20 minutes of being in bed, get out of bed instead of forcing yourself to sleep.** Engaging in a relaxing activity outside of bed such as reading a book or stretching may help you feel sleepy. Return to bed when you are sleepy again.
7. **Create a relaxing bedtime routine.** Start unwinding 1 to 2 hours before bed. A relaxing bedtime routine can include reading, journaling, or some other calming activity.
8. **Avoid engaging in stimulating or stressful activities in the evening.** Try to avoid activities that you find stressful before bed, such as watching the news or checking your email. Some useful ways to manage stress before bed include writing down thoughts that are on your mind, getting organized, and setting goals.
9. **Limit exposure to bright light in the evenings, including bright light from electronic devices.** Limit the use of electronic devices, including phones and TV, at least 30 minutes before your bedtime. Limiting light exposure tells your body that it is time to sleep. Using dimly lit lamps or light dimmer switches can help limit your exposure to bright light in the evenings. Changing the lighting settings of your electronic devices to warmer tones ("Night Shift Mode") or wearing blue-light-blocking glasses before bed can help limit your exposure to bright light at night.
10. **Empty your bladder, bowel, and ostomy bag before going to bed.** Voiding right before going to bed can help limit the number of bathroom trips you make at night.
11. **If safe and possible, limit your oral fluid intake before bedtime**. Limiting your oral fluid intake before bed can help reduce the number of bathroom trips you make at night. This may not be possible for everyone. Always discuss your oral fluid intake and IV hydration with your healthcare team before making any changes.
12. **If you eat or drink by mouth, consider earlier mealtimes, and avoid caffeine and alcohol before bed.** If you eat by mouth, aim to stop eating at least 3 hours before going to bed. This can help you fall asleep and stay asleep more easily. A light snack at bedtime is okay if you are feeling hungry. Avoid both caffeine and alcohol in the evening as they may make it more difficult for you to fall asleep and stay asleep.

Preparing your bedroom for sleep

Your bedroom environment can affect your ability to fall asleep and stay asleep. Making small changes to your bedroom can help you sleep better.

1. **Keep your bedroom cool while you sleep.** An excessively hot or cold room may make it harder for you to fall asleep and stay asleep. Aim for a bedroom that is between 60 to 68 degrees Fahrenheit or 16 to 20 degrees Celsius.
2. **Keep your bedroom as dark as safely possible.** Having a dark bedroom helps

you fall asleep and stay asleep. However, if you need some light in your bedroom or hallway, keep it as dim as possible and facing away from you. For example, floor lights can help keep your bedroom dimly lit without disrupting your sleep. If your infusion pump screen is bright, consider safely covering the screen.

3. **Make sure the area around your bed is clutter-free.** You may need to wake up in the middle of the night to use the bathroom or troubleshoot your pump. Creating a clutter-free space around your bed by having a dedicated TPN equipment area can make this quicker and safer so that you can get right back to bed.
4. **Make sure your path to the bathroom is clear.** Using the bathroom at night is often necessary. Find ways to make trips to the bathroom at night quicker and safer so that you can get right back to bed. For example, use a cart or trolley bag to help you move your pump, remove any rug or carpet that may make you fall, use night lights and dimly lit hallways, install handrails to prevent you from falling, or move your bed closer to the bathroom. Although not for everyone, if getting to the bathroom is difficult for you, a bedside commode can sometimes help.
5. **Keep your bedroom as quiet as possible.** If you find that your pump alarms are loud and disruptive, consider safely masking the noise. Some ways to do this include covering the pump, placing the pump in a pillowcase or bag, or using a white noise machine. You should still be able to hear important emergency alarms. If you use multiple pumps, try to synchronize the pumps to limit the number of alarms at night. You should not have to deal with many alarms during the night; try to find out what is causing the alarms to ring at night. If you are still struggling with too many alarms at night, speak to your healthcare team about troubleshooting or switching to a newer pump.
6. **Prepare your bed area to maximize comfort while running TPN at night.** You may experience feeling hot or cold during TPN infusions, and preparing for this can help you sleep more comfortably. For example, if you often experience night sweats, sleeping on a towel can help you fall back asleep quicker. Having an extra blanket for night chills, bringing the TPN bag to room temperature before starting your infusion, or having extra cleaning supplies within reach may help you during your sleep.
7. **If you have a bed partner, talk to them about your sleep needs.** Social support is important when receiving TPN at home, so involve your bed partner to get the help you need. For some people, it may be helpful to sleep in separate beds or rooms to avoid disrupting the sleep of others.

During the day

Certain activities and habits during the day can affect your sleep at night. Try incorporating some of the following into your daily routine to improve your sleep.

1. **Go outside and get natural sunlight for at least 30 minutes each day.** Sun exposure, especially in the morning, can help you feel awake and refreshed.
2. **Try to be active during the day to the best of your ability.** Stretching, walking,

and staying active during the day can improve your sleep. Talk to your healthcare team about being active during the day.

3. **If you benefit from napping, aim to nap within 6 to 8 hours of waking up and keep your naps to 30 minutes.** Longer naps in the evening may make it difficult to fall asleep at night. However, more frequent napping may be necessary when you are feeling unwell or recovering from surgery.

Strategies for better sleep
It may take time to find ways to improve your sleep. Here are some strategies that may help with your sleep. These strategies do not work for everyone. Through trial and error, you may be able to find something that works best for you.

1. **Try tracking your sleep for a couple of days using a sleep diary.** Tracking your sleep for at least one week can help you and your healthcare team find ways to improve your sleep and identify behaviors that may cause you to sleep poorly. You can use our sleep diary to track your sleep and TPN infusion. If you prefer to track your sleep electronically, you can also use a sleep tracking device and mobile application available for your phone. Keep in mind that sleep duration estimated by wearable technology and phone apps are not always accurate.
2. **Discuss with your healthcare team about making changes to your TPN infusion or IV hydration schedules.** Longer infusion cycles, for example 12 hours instead of 10 hours, can help reduce the number of bathroom trips at night. Shorter infusion cycles, however, can help you run your TPN quicker. Starting infusions earlier in the evening may also help by having the infusions end earlier in the morning. Discuss these possibilities, including changes to your IV hydration, with your healthcare team before making any changes.
3. **Running your TPN during the daytime can help you sleep better.** However, TPN during the daytime may be inconvenient and difficult for many. Talk to your healthcare team about switching to TPN or IV hydration during the daytime.
4. **Consider a different sleeping posture if your TPN at night is causing you discomfort.** Sleeping inclined may help relieve indigestion, reflux, and nausea.
5. **If you struggle with line tangling and mispositioning, or fear line dislodgement while sleeping, consider longer line tubing or try securing your line.** Longer line tubing or curly tubing can give you more wiggle room to shift positions at night. Using central line wraps, PICC line sleeves, or tubing clips can also help secure lines. There are also ways to minimize medical line entanglement, which can be a safety hazard, such as covering and securing lines, taping multiple lines together, fixating lines to the arms or legs, avoiding tubing near the neck area, and placing the pump under the bed.
6. **Consider replacing your IV pole with a backpack for easier movement at night.** Hanging your TPN bag on an IV pole may make it difficult for you to move at night, especially on a carpet or rug. Try using a cart, trolley bag, or backpack to help you move your pump around.
7. **Review your list of medications with your healthcare team for possible**

sleep interference. Some medications may affect how you sleep. Review your medications with your healthcare team to find which ones may be disrupting your sleep.

8. **Do you smoke tobacco or vape?** Talk to your healthcare team about ways to quit.
9. **Consider the following sleep aids that may help you with your sleep: eye masks, earplugs, blackout curtains, weighted blankets, and white noise machines.** Different aids work well for some people. Test different aids for a few nights to find out which ones may help you fall asleep and stay asleep.
10. **Create new relaxing behaviors before bed.** This can include listening to calming music or nature sounds, practicing gentle yoga, relaxing your muscles to relieve tension, wearing loose and soft clothing, and aromatherapy using essential oils.
11. **Practice meditation and mindfulness to help you relax before going to bed.** Guided meditation available on different mobile applications can help you learn new ways to control your thoughts and breathing. For example, one technique is guided imagery, in which you visualize positive images to help your body relax and relieve stress and anxiety. You can find free guided meditation resources and mobile applications on the internet.
12. **Cognitive Behavioral Therapy for Insomnia (CBT-I).** CBT-I is a common, safe, and effective treatment for people with insomnia that may be helpful if you are struggling with getting to sleep or staying asleep. Cognitive therapy, Sleep restriction therapy, Stimulus control therapy, Sleep hygiene, and Relaxation techniques are forms of CBT-I. CBT-I needs to be administered by a clinician, so please discuss this therapy with your healthcare team. You can find a trained clinician in CBT-I on the internet.
13. **Melatonin may help you fall asleep for a few nights but is unlikely to help in the long term.** Sleep medications may be useful for a few nights. Discuss sleep medications and supplements with your healthcare team before taking them.
14. **If your sleep problems continue, discuss options with your healthcare team.** Please contact your healthcare team or a sleep specialist to rule out sleep disorders such as insomnia, sleep apnea, and restless legs syndrome.

Supplementary Material 5. Sleep Diary for Consumers of Home TPN

Sleep diary for home TPN consumers

Tracking your sleep for at least one week can help you and your healthcare team find ways to improve your sleep and identify behaviors that may cause you to sleep poorly. You can use the following sleep diary to share your sleep habits with your healthcare team.

Today's Date/Current Time:______________________________

1. What time did you go to bed **last night**? **Time: ____:____ am / pm**

2. At what time did you try to fall asleep **last night**? **Time: ____:____ am / pm**

3. How long did it take you to fall asleep yesterday in minutes? **______min**

4. What time did you finally wake up **today**? **Time: ____:____ am / pm**

5. Approximately how many hours of sleep did you get at night? **_____ hour_____ min**

6. On a scale from 0 (poorly) to 10 (great), how well did you sleep **last night**? _______

7. Did you take any medications or supplements to help you sleep **last night**?
☐ **No** ☐ **Yes**
If yes, please specify: ____

8. How many times did you wake up during your sleep period? ________
List each: indicate at what time and for how long?
At what time? ______:______ am/pm For how long? _____ hour_____ min
Reason?______________________________

At what time? ______:______ am/pm For how long? _____ hour_____ min
Reason?______________________________

At what time? ______:______ am/pm For how long? _____ hour_____ min
Reason?______________________________

9. Did you nap yesterday? ☐ **No** ☐ **Yes**
If yes, how many times? __________

If yes, list each nap: indicate at what time you took a nap and for how long?
At what time? ______:______ am/pm For how long? _____ hour____ min

10. What time did you start your home TPN infusion? **Time: ____:____ am / pm**

11. What time did your home TPN infusion end? **Time: ____:____ am / pm**

12. Were there any unexpected disruptions to your TPN? ☐ **No** ☐ **Yes**
    If yes, specify at what time and for how long:
    At what time? ______:______ am/pm For how long? _____ hour_____ min

13. Did you receive any IV hydration (fluids) with your TPN? ☐ **No** ☐ **Yes**
    At what time? ______:______ am/pm For how long? _____ hour_____ min

14. Did you receive any additional lipids with your TPN? ☐ **No** ☐ **Yes**
    At what time? ______:______ am/pm For how long? _____ hour_____ min

15. Did your pump alarm go off at night? ☐ **No** ☐ **Yes**
    If yes, specify at what time:
    At what time? ______:______ am/pm For how long? _____ hour_____ min

16. Did you eat or drink anything by mouth yesterday, including coffee and alcohol?
    ☐ **No** ☐ **Yes**

If yes, please indicate here all that you have consumed (use additional pages if necessary):

Time	Location	Meal Type/ Snack	Amount/ Unit	Food/ Beverage	Preparation/ Description
7:30am	*Home*	*Breakfast*	*8 oz*	*Coffee*	*Regular – brewed*
		Breakfast	*1 Tbsp*	*HOOD half & half*	

Write here any other comments about your sleep:

Supplementary Material 6. Healthy sleep practices for consumers of home TPN (Spanish translation)

Dormir mejor al usar NPT en casa

Dormir lo suficiente y de calidad es importante para la salud física y el bienestar mental. Sin embargo, los problemas de sueño son comunes en las personas que consumen nutrición parenteral total (NPT) en casa, especialmente cuando las infusiones de NPT ocurren durante la noche mientras la persona duerme.

Los hábitos de sueño saludables que se indican más adelante en este documento pueden ayudarle a dormir mejor. La siguiente guía se basa en evidencia científica y aportes de miembros de la comunidad, entre ellos, consumidores de NPT y proveedores de atención médica. Mejorar el sueño lleva tiempo. Elija un par de cambios que usted pueda hacer basándose en la lista de abajo.

Si los problemas de sueño persisten, hable con su equipo de atención médica o con un especialista en trastornos del sueño sobre otras opciones.

Preparándose para dormir

Las cosas que usted hace antes de acostarse pueden ayudar a que el cuerpo esté preparado para dormir.

1. **Procure dormir entre 7 y 9 horas.** Un total de 7 a 9 horas de sueño es el número de horas recomendado para la mayoría de los adultos; sin embargo, es posible que usted necesite más o menos horas de sueño. Tenga en cuenta que esta cantidad de tiempo se refiere al tiempo que pasa dormido(a), y no al tiempo que pasa en la cama. Es posible que tenga que pasar más tiempo en la cama para lograr dormir de 7 a 9 horas. No afecta que este periodo de tiempo sea continuo o ininterrumpido, es común que las personas que consumen NPT se despierten varias veces en medio de la noche. Si usted se despierta con mucha frecuencia o si le cuesta volver a dormirse en medio de la noche, hable con su equipo de atención médica o con un especialista en trastornos del sueño.
2. **Procure acostarse y levantarse aproximadamente a la misma hora todos los días, incluso los fines de semana, los días en que no trabaje y las noches en que no esté consumiendo NPT.** Tener un horario de sueño fijo ayuda a regular su reloj biológico al indicarle a su cuerpo que es hora de dormir. Mantener el hábito de acostarse y levantarse a la misma hora le ayuda a quedarse dormido(a) y despertarse a la hora que usted desee.
3. **Mantenga el horario en que se pone la infusión de NPT lo más regular que le sea posible, empezando la administración siempre a la misma hora.** Ser constante con el horario en que se administra la infusión también puede ayudar a preparar al cuerpo para un mejor sueño. Sin embargo, puede ser estresante tener un horario fijo para la NPT. Hable con su equipo de atención médica sobre

la posibilidad de un horario flexible para la NPT.

4. **No se vaya a la cama hasta que esté listo(a) para dormir.** Es mejor esperar hasta estar preparado para dormir antes de acostarse en la cama. Esto le ayudará a evitar estar despierto durante mucho tiempo, lo que puede ser frustrante. Si cree que puede estar teniendo problemas de insomnio, hable con su equipo de atención médica o con un especialista en trastornos del sueño.
5. **Evite pasar demasiado tiempo en la cama.** Use su cama solo para dormir y para las relaciones íntimas. Puede haber excepciones, por ejemplo, si se siente mal o se está recuperando de una cirugía.
6. **Si lleva 20 minutos acostado(a) en la cama y no ha podido conciliar el sueño, levántese de la cama en vez de intentar dormirse a la fuerza.** Hacer una actividad relajante fuera de la cama, como leer un libro o estirarse, puede ayudar a que le dé sueño. Vuelva a acostarse cuando le dé sueño nuevamente.
7. **Establezca una rutina relajante a la hora de acostarse.** Empiece a relajarse de 1 a 2 horas antes de acostarse. Una rutina relajante antes de acostarse puede incluir leer, escribir en un diario o hacer alguna otra actividad relajante.
8. **Evite las actividades estimulantes o estresantes por la noche.** Trate de evitar actividades que le resulten estresantes antes de acostarse, como ver las noticias o mirar su correo electrónico. Algunas formas útiles de gestionar el estrés antes de dormir incluyen anotar los pensamientos que están en su mente, organizarse y establecer metas.
9. **Limite el tiempo de estar expuesto(a) a luces brillantes por las noches, incluyendo la luz brillante de los dispositivos electrónicos.** Intente dejar de usar dispositivos electrónicos, incluidos teléfonos y televisores, al menos 30 minutos antes de acostarse. Al limitar el tiempo de estar expuesto a la luz, su cuerpo entiende que es hora de dormir. Usar lámparas que emiten una luz tenue o usar reguladores de intensidad de luz (dimmer switch) puede ayudar a limitar su exposición a la luz brillante por las noches. También, cambiar la configuración de iluminación de sus dispositivos electrónicos a tonos más cálidos (“Night Shift Mode”) antes de acostarse o usar gafas que bloquean la luz azul, puede ayudar a limitar la exposición a luces brillantes en la noche.
10. **Asegúrese de evacuar los intestinos, la vejiga y la bolsa colectora para estoma antes de acostarse.** Evacuar/orinar justo antes de acostarse puede ayudar a limitar el número de veces que se levante en la noche para ir al baño.
11. **Si es posible y seguro, limite su ingesta de líquidos orales antes de acostarse.** Limitar la ingesta de líquidos orales antes de acostarse puede ayudar a reducir el número de veces que se levante en la noche para ir al baño. Esto puede no ser posible para todos. Hable siempre con su equipo de atención médica antes de hacer cualquier cambio a la ingesta de líquidos, ya sean orales o intravenosos.
12. **Si come o bebe por vía oral, intente comer más temprano y evite la cafeína y el alcohol antes de acostarse.** Si come por vía oral, intente dejar de comer al menos 3 horas antes de acostarse. Esto puede ayudarlo(a) a conciliar y mantener el sueño. Si tiene hambre, está bien comer un refrigerio liviano antes

de acostarse. Evite consumir cafeína y alcohol en la noche, ya que puede que sea más difícil conciliar el sueño y permanecer dormido(a).

Preparando su dormitorio para facilitar el sueño
El entorno de su dormitorio puede afectar su capacidad para conciliar el sueño y permanecer dormido(a). Hacer pequeños cambios en su dormitorio puede ayudarle a dormir mejor.

1. **Mantenga su dormitorio fresco mientras duerme.** Puede costarle más trabajo conciliar el sueño y permanecer dormido(a) si en su habitación hace calor o frío excesivo. Procure mantener la temperatura en su dormitorio entre 60 y 68 grados Fahrenheit o entre 16 y 20 grados Celsius.
2. **Mantenga su dormitorio lo más oscuro posible.** Mantener el dormitorio oscuro le ayuda a conciliar el sueño y permanecer dormido(a). Sin embargo, si necesita algo de luz en su dormitorio o pasillo, mantenga la luz lo más tenue posible y en sentido opuesto a usted. Por ejemplo, las lámparas de pie pueden ayudar a mantener su dormitorio poco iluminado para no interrumpir el sueño. Si la pantalla de la bomba de infusión emite una luz brillante, considere encontrar una forma segura de cubrirla.
3. **Asegúrese de que el área alrededor de su cama esté limpia y ordenada.** Puede ser que necesite levantarse en medio de la noche para usar el baño o solucionar problemas con la bomba. Tener un espacio limpio y ordenado alrededor de su cama dedicado especialmente a los equipos de NPT ayuda a que vuelva a la cama más rápidamente y de forma segura.
4. **Asegúrese de que el camino del dormitorio al baño esté libre.** A menudo es necesario usar el baño en la noche. Busque la manera de hacer que las visitas al baño por la noche sean más rápidas y seguras para que pueda volver a acostarse enseguida. Por ejemplo, use un carrito o una maleta con ruedas para ayudarse a mover la bomba, retire cualquier alfombra o tapete que pueda causar caídas, use lamparillas de luz tenue y mantenga baja la iluminación en los pasillos, instale pasamanos para evitar caídas o coloque su cama más cerca del baño. Aunque no son aptos para todos, los inodoros portátiles al lado de la cama a veces pueden ayudar si le resulta difícil llegar al baño.
5. **Mantenga su dormitorio lo más silencioso posible.** Si cree que las alarmas de su bomba son ruidosas y molestas, considere una forma segura de amortiguar el ruido. Algunas formas de hacerlo incluyen cubrir la bomba, colocarla en una funda de almohada o bolsa, o usar una máquina de ruido blanco. Igualmente debería poder escuchar las alarmas de emergencia que son importantes. Si utiliza varias bombas, intente sincronizar las bombas para limitar el número de alarmas por la noche. No debería tener que lidiar con muchas alarmas durante la noche; intente averiguar qué está causando que las alarmas suenen por la noche. Si todavía tiene demasiadas alarmas por la noche, hable con su equipo de atención médica sobre cómo solucionar el problema o sobre la posibilidad de cambiar su bomba por una más nueva.

6. **Prepare la cama para maximizar su comodidad mientras se administra la NPT por la noche.** Es posible que sienta calor o frío durante las infusiones de NPT, y estar preparado(a) para esta posibilidad puede ayudarle a dormir más cómodamente. Por ejemplo, si usted suda a menudo durante la noche, dormir sobre una toalla puede ayudarlo(a) a volver a dormirse más rápidamente. También para ayudarle durante el sueño, puede tener una manta adicional por si tiene frío durante la noche, puede poner la bolsa de NPT a temperatura ambiente antes de comenzar la infusión, o puede tener productos de limpieza adicionales a mano.
7. **Si alguien más duerme con usted en la misma cama, hable con él/ella sobre lo que usted necesita para dormir.** El apoyo social es importante cuando una persona recibe NPT en casa, así que involucre a su compañero(a) de cama para que le brinde la ayuda que usted necesite. Para algunas personas, puede ser útil dormir en camas o habitaciones separadas para evitar interrumpir el sueño de los demás.

Durante el día mejor
Ciertas actividades y hábitos durante el día pueden afectar el sueño durante la noche. Intente incorporar algunos de los siguientes hábitos en su rutina diaria para mejorar el sueño.

1. **Salga al aire libre para tomar un poco de sol durante al menos 30 minutos al día.** Exponerse al sol, especialmente por la mañana, puede ayudarle a sentirse despierto(a) y renovado(a).
2. **Intente estar activo(a) durante el día lo más que pueda.** Estirarse, caminar y mantenerse activo(a) durante el día pueden mejorar el sueño. Hable con su equipo de atención médica sobre estar activo(a) durante el día.
3. **Si usted generalmente duerme la siesta, intente hacerlo de 6 a 8 horas después de la hora en que se despierta y limite las siestas a 30 minutos.** Las siestas prolongadas por la tarde pueden hacer que sea difícil conciliar el sueño en la noche. Sin embargo, puede ser necesario dormir siestas con más frecuencia cuando se sienta mal o se esté recuperando de una cirugía.

Estrategias para dormir mejor
Puede llevar tiempo encontrar formas de mejorar el sueño. Estas son algunas estrategias que pueden ayudarle a dormir mejor. Estas estrategias no funcionan para todos(as). Por ensayo y error, puede que encuentre lo que funcione mejor para usted.

1. **Utilice un diario de sueño e intente llevar un registro del sueño durante un par de días.** Llevar un registro del sueño durante al menos una semana puede ayudarle a usted y a su equipo de atención médica a encontrar formas de mejorar el sueño e identificar comportamientos que pueden hacer que duerma mal. Puede usar nuestro diario de sueño para llevar un registro del sueño y de la infusión de NPT. Si prefiere llevar un registro electrónico del sueño, también

puede utilizar un dispositivo de seguimiento del sueño o una aplicación móvil disponible en su teléfono. Tenga en cuenta que la tecnología portátil y las aplicaciones para teléfonos inteligentes no siempre dan medidas precisas de la duración del sueño.

2. **Hable con su equipo de atención médica sobre la posibilidad de cambiar los horarios de infusión de la NPT o de la hidratación intravenosa.** Los ciclos de infusión más prolongados, por ejemplo, 12 horas en lugar de 10 horas, pueden ayudar a reducir el número de visitas al baño por la noche. Además, los ciclos de infusión más cortos pueden ayudarle a administrar la NPT más rápidamente. Comenzar las infusiones más temprano en la tarde también puede ayudar a que finalicen más temprano en la mañana. Hable con su equipo de atención médica sobre estas posibilidades antes de hacer cualquier cambio, incluyendo cualquier ajuste a su hidratación intravenosa.
3. **Administrarse la NPT durante el día puede ayudarle a dormir mejor.** Sin embargo, administrarse la NPT durante el día puede ser inconveniente y difícil para muchas personas. Hable con su equipo de atención médica sobre la posibilidad de cambiar la NPT y la hidratación intravenosa a un horario diurno.
4. **Considere una posición diferente para dormir si siente molestias al administrarse la NPT en la noche.** Dormir elevado(a) puede ayudar a aliviar la indigestión, el reflujo y las náuseas.
5. **Si se le enreda el catéter, se le mueve a una posición incorrecta, o si teme que se pueda salir mientras duerme, considere una vía más larga o intente sujetarla.** Los catéteres más largos o los catéteres en espiral pueden darle más libertad para cambiar de posición durante la noche. Utilizar cubiertas para catéteres centrales, mangas para catéteres de tipo PICC o clips de tubería también puede ayudar a mantener las vías sujetas. Los enredos en las vías pueden peligrar su seguridad, por esa razón existen estrategias para minimizarlos, como cubrir y sujetar las vías, juntar varias vías con cinta adhesiva, fijarse las vías a los brazos o las piernas, no colocar tubos cerca del área del cuello y colocar la bomba debajo de la cama.
6. **Considere cambiar su portasuero por una mochila para facilitar el movimiento por la noche.** Colgar su bolsa de NPT en un portasueros puede dificultar su movimiento durante la noche, especialmente sobre alfombras o tapetes. En lugar de eso, utilice un carrito, una maleta con ruedas o una mochila para mover la bomba.
7. **Revise su lista de medicamentos con su equipo de atención médica para determinar si alguna puede estar interfiriendo con su sueño.** Algunos medicamentos pueden afectar el sueño. Revise sus medicamentos con su equipo de atención médica para determinar cuáles pueden estar perturbando su sueño.
8. **¿Fumas tabaco o vapeas?** Hable con su equipo de atención médica sobre formas para dejar de fumar.
9. **Considere las siguientes cosas para ayudar a mejorar el sueño: antifaz para dormir, tapones para los oídos, cortinas opacas, mantas con peso y**

máquinas de ruido blanco. Ciertas funcionan mejor para algunas personas sobre otras. Pruebe diferentes cambios durante algunas noches para averiguar cuáles pueden ayudarle a conciliar el sueño y permanecer dormido(a).

10. **Practique nuevos comportamientos relajantes antes de acostarse.** Esto puede incluir escuchar música relajante o sonidos de la naturaleza, practicar yoga suave, relajar los músculos para aliviar la tensión, usar ropa holgada y suave, y aromaterapia con aceites esenciales.
11. **Practique meditación y atención plena (mindfulness) para relajarse antes de acostarse.** La meditación guiada disponible en diferentes aplicaciones móviles puede ayudarle a aprender nuevas formas de controlar los pensamientos y la respiración. Por ejemplo, una técnica es la visualización o imaginación guiada cuyo objetivo es visualizar imágenes o escenarios positivos que ayudan a relajar el cuerpo, y aliviar el estrés y la ansiedad. Puede encontrar recursos gratuitos de meditación guiada y aplicaciones móviles en internet.
12. **Terapia Cognitiva Conductual para el insomnio (TCC-I).** TCC-I es un tratamiento común, seguro y eficaz a largo plazo para personas con insomnio que le puede ser útil si tiene dificultades para conciliar el sueño o permanecer dormido(a). La terapia cognitiva, la restricción del sueño, el control de estímulos, la higiene del sueño y las técnicas de relajación son formas de TCC-I. La TCC-I necesita ser administrada por un clínico especializado, así que hable sobre esta terapia con su equipo de atención médica. Puede encontrar un proveedor clínico capacitado en TCC-I en internet.
13. **La melatonina puede ayudarle a conciliar el sueño durante algunas noches, pero es poco probable que ayude a largo plazo.** Los medicamentos para el sueño pueden ser útiles durante algunas noches. Hable sobre medicamentos y suplementos para dormir con su equipo de atención médica antes de tomarlos.
14. **Si los problemas de sueño continúan, considere hablar sobre otras opciones con su equipo de atención médica.** Póngase en contacto con su equipo de atención médica o con un especialista en trastornos del sueño para descartar posibles trastornos como insomnio, apnea del sueño o síndrome de piernas inquietas.

Supplementary Material 7. Sleep Diary for Consumers of Home TPN (Spanish translation)

Diario de sueño para consumidores de NPT en el hogar

Llevar un registro del sueño durante al menos una semana puede ayudarle a usted y a su equipo de atención médica a encontrar formas de mejorar su sueño. Puede usar el siguiente diario para compartir sus hábitos de sueño con su equipo de atención médica.

Fecha/hora de hoy: ______________________________

1. ¿A qué hora se acostó **anoche**? **Hora: ____:____ am / pm**

2. ¿A qué hora intentó quedarse dormido(a) **anoche**? **Hora: ____:____ am / pm**

3. ¿Cuánto tiempo (en minutos) tardó en dormirse ayer? **______min**

4. ¿A qué hora se despertó hoy finalmente? **Hora: ____:____ am / pm**

5. Aproximadamente, ¿cuántas horas durmió durante la noche? **_____ hora ____ min**

6. En una escala del 0 (mal) al 10 (excelente), ¿qué tan bien durmió **anoche**? _______

7. ¿Tomó algún medicamento o suplemento para dormir **anoche**? ☐ **No** ☐ **Sí**
   En caso afirmativo, por favor, especifique: __________________________

8. ¿Cuántas veces se despertó mientras dormía? ________
   Enumere cada ocasión: ¿indique a qué hora y por cuánto tiempo?
   ¿A qué hora? ______:______ am/pm ¿Por cuánto tiempo? _____ hora_____ min
   ¿Razón?______________________________

   ¿A qué hora? ______:______ am/pm ¿Por cuánto tiempo? _____ hora_____ min
   ¿Razón?______________________________

   ¿A qué hora? ______:______ am/pm ¿Por cuánto tiempo? _____ hora_____ min
   ¿Razón?______________________________

9. ¿Durmió siesta ayer? ☐ **No** ☐ **Sí**
   En caso afirmativo, ¿cuántas veces? __________

En caso afirmativo, enumere cada siesta: indique a qué hora durmió la siesta y por cuánto tiempo.
¿A qué hora? ______:______ am/pm ¿Por cuánto tiempo? _____ hora____ min

10. ¿A qué hora comenzó su infusión de NPT en casa? **Time: ____:____ am / pm**

11. ¿A qué hora terminó su infusión de NPT en casa? **Time: ____:____ am / pm**

12. ¿Ocurrió alguna interrupción imprevista mientras se administraba la NPT? ☐ **No** ☐ **Sí**
En caso afirmativo, especifique a qué hora y durante cuánto tiempo:
¿A qué hora? ______:______ am/pm ¿Por cuánto tiempo? _____ hora_____ min

13. ¿Recibió hidratación intravenosa (líquidos) con la NPT? ☐ **No** ☐ **Sí**
¿A qué hora? ______:______ am/pm ¿Por cuánto tiempo? _____ hora_____ min

14. ¿Recibió algún lípido adicional con la NPT? ☐ **No** ☐ **Sí**
¿A qué hora? ______:______ am/pm ¿Por cuánto tiempo? _____ hora_____ min

15. ¿La alarma de la bomba sonó por la noche? ☐ **No** ☐ **Sí**
En caso afirmativo, especifique a qué hora:
¿A qué hora? ______:______ am/pm ¿Por cuánto tiempo? _____ hora_____ min

16. ¿Comió o bebió algo por vía oral ayer, incluyendo café y alcohol?
☐ **No** ☐ **Sí**

En caso afirmativo, indique aquí todo lo que consumió (use páginas adicionales si es necesario):

**Hora**	**Lugar**	**Tipo de comida/ refrigerio (snack)**	**Cantidad /Unidad**	**Comida/Bebida**	**Preparación/Descripción**

7:30am	*En casa*	*Desayuno*	*8 oz*	*Café*	*Normal– preparado en casa*
		Desayuno	1 cda.	*HOOD half & half (mitad crema, mitad leche HOOD)*	

Escriba aquí cualquier otro comentario sobre su sueño:

Supplementary Table 1. Synthesis of Sleep Health Recommendations for Consumers of Home Parenteral Nutrition

Theme	Recommendation	Target Population	Source(s)	Relevance to HPN Community (Yes/No)	Practicality to HPN Community
Recommended sleep duration	"Adults should sleep 7 or more hours per night on a regular basis to promote optimal health."	Healthy adults aged 18-60 years	American Academy of Sleep Medicine (AASM): Watson NF, Badr MS, Belenky G, et al. Recommended Amount of Sleep for a Healthy Adult: A Joint Consensus Statement of the American Academy of Sleep Medicine and Sleep Research Society. Sleep. 2015;38(6):843-844. Published 2015 Jun 1. doi:10.5665/sleep.4716	Yes	Be explicit - make distinction between time in bed & actual sleep hours, and include upper bound (TIB is not actually TST). Be mindful of language (ideal>should). Cycle may exceed ideal sleep duration; important consideration to bring up in focus groups.<br><br>Sleep is not continuous due to sleep interruptions (have to be mindful & conscious when awake ~1-2 hrs, beyond HPN bag), so 7-9 hr block may not be attainable. Can split up in recommendation; phrase as 7-9 hrs total sleep time; this duration doesn't have to be continuous/uninterrupted.
Consistency and routine	"Be consistent. Go to bed at the same time each night and get up at the same time each morning, including on the weekends."	General, insomnia	Centers for Disease Control & Prevention (CDC)	Yes	Infusion time may help regulate consistent bedtime routine, especially relevant to those who don't run every night.<br><br>Tie to consistent TPN infusion times, for example: maintain consistent sleep-wake schedule even if not infusing that night (rather than change how HPN is prescribed & infusion schedule - address in clinician focus groups).
	"Establish a relaxing bedtime routine."	General, insomnia	American Academy of Sleep Medicine (AASM)	Yes	Be more explicit about what this entails, for example warm bath problematic for central line; tea only if able to eat or drink; tea also contradicts w/ avoiding liquids before bed. Perhaps no electronics or blue light; watching TV.
Environment	"Make sure your bedroom is quiet, dark, relaxing, and at a comfortable temperature."	General	Centers for Disease Control & Prevention (CDC)	Yes	May not be entirely practical because of loud pumps, unless tuned out. Issues w/ perceived safety if too dark & quiet. Dark room is not always feasible; lines & bags need to be visible (some light but not direct); can recommend that light be made dim & face away (ex. under counter light, LED light, amber light wavelength, night light). As dark & quiet as possible; stay away from rigidity due to complexity.<br><br>Specify ideal temperature and be cognizant of temp range (may have to widen); people will run hot or cold when on TPN.
	Noise reduction or auditory masking<br>"Masking of noise has been demonstrated to improve sleep quantity and to reduce the number of nighttime awakenings"	ICU, critically ill	Pulak LM, Jensen L. Sleep in the Intensive Care Unit: A review. J Intensive Care Med. 2016;31(1):14-23. doi:10.1177/0885066614538749	Yes (but safety concern)	Have to address pump alarms; may alert if movement or tubing issue. Pumps necessary to avoid missing a problem or concern. May be okay as long as pump is still audible (which it often is). Manufacturers may play role in adapting pumps: turning pump on or off w/ voice activation; vibrating; volume control (minimal sound).<br><br>Consider: mechanical noise/sound machine is not necessarily silence.
	"Barrier free access to a toilet or toilet chair"	Nocturia, nocturnal polyuria (NP)	Weiss JP, Everaert K. Management of Nocturia and Nocturnal Polyuria. Urology. 2019;133S:24-33. doi:10.1016/j.urology.2019.09.022	Yes, frequent complaint	May not reduce wake after sleep onset but a barrier-free path is important for safety. Decreasing barriers to toilet may be more practical than devices (ex. catheter, commode). Sleep closest to restroom, minimize rug or carpet to avoid tripping/falls. Commode is a potential solution, but familiarity of bathroom might be easier; could be useful post surgery due to limited mobility; depends on person so bring up in focus groups. Be cognizant of IV poles (wheels won't move on carpet) devices can be dependent on preference.
	"Install night lights to illuminate your path to the bathroom."	Nocturia, nocturnal polyuria (NP)	Harvard Health	Yes	*Potential strategy, similar to other recs in this same category*
	"Keep pathways free of trip hazards such as electrical cords, throw rugs, and loose carpeting."	Nocturia, nocturnal polyuria (NP)	Harvard Health	Yes	*Potential strategy, similar to other recs in this same category*
	"Install handrails in hallways and grab bars in bathrooms."	Nocturia, nocturnal polyuria (NP)	Harvard Health	Yes	*Potential strategy, similar to other recs in this same category*
	"Consider using a bedside commode if it's hard to get to the bathroom safely."	Nocturia, nocturnal polyuria (NP)	Harvard Health	Yes	*Potential strategy, similar to other recs in this same category*
	"Helpful sleep aids include blackout curtains, eye masks, weighted blankets, white noise machines and ear plugs."	Narcolepsy	Wake Up Narcolepsy	Yes	Earplugs & eye masks can be irritating for some; earplugs can also block out too much noise and might not hear done beep (be careful here); practical but may depend on the person.
	"Get rid of sound and light distractions in your bedroom during your daytime sleep. "	Night shift workers	National Heart, Lung, and Blood Institute (NHLBI)	No	May be relevant to night shift workers; non pump related noises
	"Keep sheets clean, neatly tucked in, and as free from wrinkles as possible."	Cancer	American Cancer Society	No	Remove tucked in sheet; can be obstruction at night to void.
	"Ask caregivers to plan care so they wake you up the least number of times during the night."	Hospitalized, cancer patients	NIH National Cancer Institute	No	Remove
	"Forest therapy: The natural environment can be used as a therapeutic resource for emotional relaxation and stress management for people who live in cities."	Cancer	Acker KA, Carter P. Sleep-Wake Disturbances in Oncology. Nursing Clinics of North America. 2021;56;175-187. doi: 10.1016/j.cnur.2021.03.001.<br><br>Kim H, Lee YW, Ju HJ, Jang BJ, Kim YI. An Exploratory Study on the Effects of Forest Therapy on Sleep Quality in Patients with Gastrointestinal Tract Cancers. Int J Environ Res Public Health. 2019;16(14):2449. Published 2019 Jul 10. doi:10.3390/ijerph16142449	Yes	Feasibility of this is unclear; tie into relaxation before bed (mindfulness meditation) and reducing barriers.<br><br>Recorded rain sounds; nature sounds; more feasible; mindfulness meditation apps; relaxing natural surroundings within bedroom (ex. wood, fake plants, driftwood), circadian lighting (Amazon). Create section on preparing sleep environment.
Electronic devices	"Remove electronic devices, such as TVs, computers, and smart phones, from the bedroom."	General	Centers for Disease Control & Prevention (CDC)	Yes	Safety concern; getting help from caregivers. If logging (input/output) - may need to keep within arm's reach. If it needs to be in arm's reach, change settings (ex. Night shift mode) and limit light exposure. Limit exposure to electronic devices or limit exposure to stimulating activities in the evening (some find phones help them sleep).<br><br>These can't be individualized so "avoid stimulating behaviors" offers more nuanced approach; block out external stimuli, don't use if arousing. Be flexible and avoid rigidity.
	"Turn off electronic devices at least 30 minutes before bedtime."	General	AASM	Yes	Combine w/ previous; ideally keep out of the room but if can't, limit use at least 30 min before bedtime (prevent arousal).
Consumption before bed	"Avoid large meals, caffeine, and alcohol before bedtime"	General, applies to many pt populations	Centers for Disease Control & Prevention (CDC)	Yes, if you eat	Frame as general w/ exceptions (for those who eat); be explicit about diuretics (alcohol & caffeine).
	"Reduce your fluid intake before bedtime."	General, nocturia	AASM	Yes	Consider fluids and oral rehydration therapy. Avoid any liquid before bed but note exceptions: ex. drinking fluid to thin output, milk for stomach acid; talk to team.
	"Don't eat a large meal before bedtime. If you are hungry at night, eat a light, healthy snack."	General	AASM	Yes, if you eat	Avoid digestive upsets/unneeded that might wake you up while asleep.
	"Emptying the bladder before going to bed"	Nocturia, nocturnal polyuria (NP)	Weiss JP, Everaert K. Management of Nocturia and Nocturnal Polyuria. Urology. 2019;133S:24-33. doi:10.1016/j.urology.2019.09.022	Yes	Combine w/ emptying ostomy bag.
	"Reducing dietary salt intake"	Nocturia, nocturnal polyuria (NP)	Weiss JP, Everaert K. Management of Nocturia and Nocturnal Polyuria. Urology. 2019;133S:24-33. doi:10.1016/j.urology.2019.09.022	No	Remove: in conflict w/ nutrition guidelines.
	"Restricting total fluid consumption to <2 L/day, if comorbidities allow"	Nocturia	Oelke M, De Wachter S, Drake MJ, et al. A practical approach to the management of nocturia. Int J Clin Pract. 2017;71(11):e13027. doi:10.1111/ijcp.13027	No	Remove: depends on person.
	"Eat more high-fiber foods during the day."	Cancer	NIH National Cancer Institute	No	May conflict w/ nutrition prescription.
	"Eat a high-protein snack (such as milk or turkey) 2 hours before bedtime."	Cancer	NIH National Cancer Institute	No	
	"Avoid heavy, spicy, or sugary foods before bedtime."	Cancer	NIH National Cancer Institute	No	
	"Empty your bowel and bladder before going to bed."	Cancer	NIH National Cancer Institute	Yes	Empty ostomy bag & bladder; good practice before bed.

**Physical activity and weight**	**"Get some exercise. Being physically active during the day can help you fall asleep more easily at night."**	General, insomnia, nocturia, depression/anxiety	Centers for Disease Control & Prevention (CDC)	Yes	Restricted so coordinate w/ team what you are able to do. Communicate w/ team abt being physically active, esp if weight is shifting; ex. Light walks, avoid lifting heavy.<br><br>Calorie intake vs. expenditure - be cognizant; may conflict w/ caloric needs; may not want to be too active & lose too much weight.
	**"Lose weight if you are overweight."**	Sleep apnea	NHLBI	No	Low prevalence of sleep apnea among HPN pts. Nutritional prescriptions manage weight so this may not be a concern
**Time in bed**	**"Use your bed only for sleep and sex."**	General, insomnia	AASM	Yes	Exceptions: illness. Postsurgery may influence time spend in bed.
	**"Don't go to bed unless you are sleepy."**	General, insomnia	AASM	Yes	If insomnia, consider sleep treatment w/ behavioral specialist.<br><br>Limit time in bed - ask how do they spend those hours prior to sleep? Where do they spend them, do they go to bed earlier than they actually do? If tapering before bed too early (esp for long infusions), maybe recommend not doing so in bed.
	**"If you don't fall asleep after 20 minutes, get out of bed. Go do a quiet activity without a lot of light exposure. It is especially important to not get on electronics."**	General, insomnia	AASM	Yes	Can reduce anxiety to complete an activity if trouble falling asleep.
	**Sleep restriction therapy (paradoxical intention): "Uses a paradoxical approach in which the patient spends less time in bed (by associating time spent in bed with time spent sleeping)"**	Insomnia	Ramakrishnan K, Scheid DC. Treatment options for insomnia. Am Fam Physician. 2007;76(4):517-526.	No	Dangerous; should be done w/ hc professional.
**Light exposure**	**"Limit exposure to bright light in the evenings."**	General	AASM	Yes	As mentioned earlier, use warm lights and dim lights before bed.
	**"Try to get outside in natural sunlight for at least 30 minutes each day"**	General, insomnia	NHLBI	Yes	Lot of time spent indoors and sometimes we need natural light; talk abt circadian shifts.
	**"When it’s time to wake up, let in as much natural light as possible."**	Narcolepsy	Wake Up Narcolepsy	Yes	
	**"Exposure to bright light on the job can improve alertness during night shift work."**	Shift workers	AASM	No	Mostly relevant for shift workers on HPN.
	**"Use dim red lights for night lights. Red light is less likely to shift circadian rhythm and suppress melatonin."**	General	Harvard Health	Yes	Controlling light temperature (LED lights) & spectrum.
	**"Consider wearing blue-blocking glasses or installing an app that filters the blue/green wavelength at night."**	General	Harvard Health	Yes	
	**"Restrict the light spectrum to wavelengths that are less likely to suppress melatonin in humans." (red instead of blue)**	ICU, critically ill, hospitalized, pts facing light-at-night exposure	Nelson RJ, DeVries AC. Medical hypothesis: Light at night is a factor worth considering in critical care units. Adv Integr Med. 2017;4(3):115-120. doi:10.1016/j.aimed.2017.12.001<br><br>Figueiro MG, Bierman A, Plitnick B, Rea MS. Preliminary evidence that both blue and red light can induce alertness at night. BMC Neurosci. 2009;10:105. Published 2009 Aug 27. doi:10.1186/1471-2202-10-105	Yes	
**Daytime napping**	**"Limit naps to no more than one hour and avoid napping late in the day."**	General, insomnia	Mayo Clinic	Yes	May depend on the person; 30 minutes or less; consider difficult time napping. Limit daytime nap whenever possible; keep short & early. Can be difficult since many on TPN don't work; less routine.
	**"If possible, plan to take a 20-30 minute nap during a break in your shift to improve alertness without grogginess"**	Shift workers	AASM	No	Mostly relevant for shift workers on HPN.
	**"If possible, nap somewhere other than your bed"**	Cancer	NIH National Cancer Institute	Yes	Create tips on how to take an effective nap; environment (light, noise, comfort), time, how to wake up.
**Medications (interference)**	**"Avoid medicines that delay or disrupt your sleep, if possible."**	General	National Heart, Lung, & Blood Institute	Yes	Medication interference very personalized; review medications w/ healthcare professional for interference. Avoiding meds that disrupt sleep, such as steroids & diuretics first thing in the morning. Not always alternatives for HPN if absorption issues.<br><br>Talk to your doc about medications for sleep; visit sleep center for sleep problems; screenings for meds, snoring, stimulants.
	**"Try to avoid taking sleeping pills."**	General	Harvard	No	Remove
	**"For those on diuretics, these could be taken midafternoon rather than prior to retiring, considering the half-life of the specific diuretic"**	Nocturia due to nocturnal polyuria (NP)	Weiss JP, Everaert K. Management of Nocturia and Nocturnal Polyuria. Urology. 2019;133S:24-33. doi:10.1016/j.urology.2019.09.022	Yes	Review medications w/ healthcare professional for interference
	**"Avoid sedative medications such as sleeping pills"**	Sleep apnea	American Lung Association	No	Remove; avoid sleeping pills, sedatives
	**"Take your medications at the same time every day, and make sure your health care team knows the schedule for them."**	Cancer	American Cancer Society	Yes	Rather than taking at same time every day, make healthcare team aware of schedule
**Smoking**	**"If you use tobacco in any form, quit."**	General	Harvard	Yes	Relevant for sleep promotion due to association of smoking with lower sleep duration. We see previous smokers in our cohorts. Include vaping.
	**"Quit smoking"**	Sleep apnea	American Lung Association	Yes	Consider recommending a smoking cessation program; combine with previous recommendation.
	**Cognitive Behavioral Therapy (CBT): "Helps change incorrect beliefs and attitudes about sleep (e.g., unrealistic expectations, misconceptions, amplifying consequences of sleeplessness); techniques include reattribution training (i.e., goal setting and planning coping responses), decatastrophizing (aimed at balancing anxious automatic thoughts), reappraisal, and attention shifting"**	Insomnia (CBTI), cancer, critically ill, PTSD/nightmares, elderly, anxiety, depression, hot flashes	Ramakrishnan K, Scheid DC. Treatment options for insomnia. Am Fam Physician. 2007;76(4):517-526.<br><br>Edinger JD, Arnedt JT, Bertisch SM, et al. Behavioral and psychological treatments for chronic insomnia disorder in adults: an American Academy of Sleep Medicine systematic review, meta-analysis, and GRADE assessment. J Clin Sleep Med. 2021;17(2):263-298. doi:10.5664/jcsm.8988	Yes	Define CBT-I as a treatment for chronic insomnia and outline structure since it will need to be led by a behavioral specialist. Perhaps a blanket statement that if insomnia is suspected, to see a sleep physician and also to seek out behavioral treatments for insomnia.
	**Brief behavioral therapy (BBT): "Due to financial constraints and lack of psychological resources needed for CBT-I, a shorter form of therapy known as brief behavioral therapy for insomnia is also available and involves core techniques from CBT-I, directed at improving circadian regulation of sleep in more than two sessions."**	Insomnia, elderly	Patel D, Steinberg J, Patel P. Insomnia in the Elderly: A Review. J Clin Sleep Med. 2018;14(6):1017-1024. Published 2018 Jun 15. doi:10.5664/jcsm.7172<br><br>Buysse DJ, Germain A, Moul DE, et al. Efficacy of Brief Behavioral Treatment for Chronic Insomnia in Older Adults. Arch Intern Med. 2011;171(10):887–895. doi:10.1001/archinternmed.2010.535	Yes	Too specific as we are describing treatment and not providing. Place under CBT umbrella recommendation.

Behavioral therapy	**Stimulus control therapy: "A set of instructions designed to (1) extinguish the association between the bed/bedroom and wakefulness to restore the association of bed/bedroom with sleep, and (2) establish a consistent wake-time."**	Insomnia	Edinger JD, Arnedt JT, Bertisch SM, et al. Behavioral and psychological treatments for chronic insomnia disorder in adults: an American Academy of Sleep Medicine systematic review, meta-analysis, and GRADE assessment. J Clin Sleep Med. 2021;17(2):263-298. doi:10.5664/jcsm.8988	No	Too specific as we are describing treatment and not providing. Place under CBT umbrella recommendation.
	**Temporal control therapy "Focuses on reestablishing a constant sleep-wake cycle. The patient is instructed to wake up at the same time daily, regardless of how much he or she slept during the night, and to avoid daytime naps"**	Insomnia	Maness DL, Khan M. Nonpharmacologic management of chronic insomnia. Am Fam Physician. 2015;92(12):1058-1064.	Yes	Relevant but mentioned previously (consistency), combine with consistency recommendation.
	"**Stress management** might help. Start with the basics, such as getting organized, setting priorities and delegating tasks. Meditation also can ease anxiety."	General, insomnia	Mayo Clinic	Yes	Phrase as avoiding stress at night, as it is a potential cause of sleep disruption.
	**Mindfulness approaches: "used as a form of meditation emphasizing a nonjudgmental state of heightened or complete awareness of one's thoughts, emotions, or experiences on a moment-to-moment basis"**	Insomnia	Edinger JD, Arnedt JT, Bertisch SM, et al. Behavioral and psychological treatments for chronic insomnia disorder in adults: an American Academy of Sleep Medicine systematic review, meta-analysis, and GRADE assessment. J Clin Sleep Med. 2021;17(2):263-298. doi:10.5664/jcsm.8988	Yes	Group with stress management; mindfulness and meditation as part of CBT. Support groups are common in the TPN community.
	**Brief therapies for insomnia (BTIs) "abbreviated versions of CBT-I (typically 1–4 sessions) emphasizing the behavioral components."**	Insomnia	Edinger JD, Arnedt JT, Bertisch SM, et al. Behavioral and psychological treatments for chronic insomnia disorder in adults: an American Academy of Sleep Medicine systematic review, meta-analysis, and GRADE assessment. J Clin Sleep Med. 2021;17(2):263-298. doi:10.5664/jcsm.8988	Yes	Relevant, but too specific as we are describing treatment and not providing; combine with CBT.
	**"Keep a sleep diary"**	Cancer	American Cancer Society	Yes	Practical especially to promote sleep consistency, but consider that this population already has to do a lot of diaries (food, urine, outputs). Also specific and is a CBT-I treatment so combine with existing.
	**Guided imagery "A technique in which a person visualizes positive images or scenarios in their mind. During guided imagery, a person uses all their senses in their imagination to help relax the body and develop a sense of well-being."**	Cancer, anxiety	NIH National Cancer Institute	Yes	Practical but related to mindfulness, so group together.
	**Self hypnosis: "A trance-like state in which a person becomes more aware and focused on particular thoughts, feelings, images, sensations, or behaviors. While under hypnosis, a person may feel calm, relaxed, and more open to suggestion."**	Cancer	NIH National Cancer Institute	No	Other forms of CBT may be sufficient and people may not trust the concept; remove.
	**CBT for CPAP "several studies have employed cognitive behavioral therapy (CBT) as intervention strategies with some success...studies suggest that targeting psychosocial factors with interventions to promote CPAP use are likely effective."**	CPAP-wearing sleep apnea patients	Sawyer AM, Gooneratne NS, Marcus CL, Ofer D, Richards KC, Weaver TE. A systematic review of CPAP adherence across age groups: clinical and empiric insights for developing CPAP adherence interventions. Sleep Med Rev. 2011;15(6):343-356. doi:10.1016/j.smrv.2011.01.003	No	Remove as we see low prevalence of apnea in this population.
	**"Involve your bed partner"**	CPAP-wearing sleep apnea patients	SleepApnea.org	No	Remove as we see low prevalence of apnea in this population.
	**Image rehearsal therapy "is recommended for the treatment of PTSD-associated nightmares and nightmare disorder" (AASM).**	PTSD associated nightmares, nightmare disorders	Morgenthaler TI, Auerbach S, Casey KR, et al. Position Paper for the Treatment of Nightmare Disorder in Adults: An American Academy of Sleep Medicine Position Paper. J Clin Sleep Med. 2018;14(6):1041-1055. Published 2018 Jun 15. doi:10.5664/jcsm.7178	No	CBT component is relevant, but consolidate with CBT-I.
	**Benson's relaxation response: a focused relaxation method that has resulted in improved self-reported sleep quality. "Consists of mindfulness techniques which affects many physical and psychological symptoms such as anxiety, pain, depression, and mood."**	Cancer	Acker KA, Carter P. Sleep-Wake Disturbances in Oncology. Nursing Clinics of North America. 2021;56;175-187. doi: 10.1016/j.cnur.2021.03.001.<br><br>Harorani M, Davodabady F, Farahani Z, Ali Kh, Rafiei F. The effect of Benson's relaxation response on sleep quality and anorexia in cancer patients undergoing chemotherapy: A randomized controlled trial. Complement Ther Med. 2020;50. doi: 10.1016/j.ctim.2020.102344.	No	Too specific; other forms of CBT mentioned above should suffice.
	**Paradoxical intention therapy: "The patient is instructed to remain awake as long as possible after getting into bed. The patient is instructed to purposefully engage in the feared activity (staying awake) to reduce performance anxiety and conscious intent to sleep that confound associated goal-directed behavior (falling asleep). This method alleviates both the patient's excessive focus on sleep and anxiety over not sleeping; as a result, sleep becomes less difficult to initiate."**	Insomnia	Edinger JD, Arnedt JT, Bertisch SM, et al. Behavioral and psychological treatments for chronic insomnia disorder in adults: an American Academy of Sleep Medicine systematic review, meta-analysis, and GRADE assessment. J Clin Sleep Med. 2021;17(2):263-298. doi:10.5664/jcsm.8988<br><br>Jansson-Fröjmark M, Alfonsson S, Bohman B, Rozental A, Norell-Clarke A. Paradoxical intention for insomnia: A systematic review and meta-analysis. J Sleep Res. 2022;31(2):e13464. doi:10.1111/jsr.13464	No	Remove as other therapies are mentioned above.

	**Hypnotics:**<br>**"recommended when immediate symptom response is desired, when insomnia produces serious impairment, when nonpharmacologic measures do not produce the desired improvement, or when insomnia persists after treatment of an underlying medical condition."**	Insomnia, cancer-related insomnia, sleep disorders	Ramakrishnan K, Scheid DC. Treatment options for insomnia. Am Fam Physician. 2007;76(4):517-526.<br><br>Sateia MJ, Buysse DJ, Krystal AD, Neubauer DN, Heald JL. Clinical practice guideline for the pharmacologic treatment of chronic insomnia in adults: an American Academy of Sleep Medicine clinical practice guideline. J Clin Sleep Med. 2017;13(2):307–349.	No	Recommend a review of all medications being taken; perhaps a blanket statement about discussing with your doctor. However do not include pharmacologic measures in our handout.
	**Benzodiazepines:**<br>**"effective for treating chronic insomnia but have significant adverse effects and the risk of dependency...benzodiazepines are most useful for short-term treatment;**<br>**however, long-term use may lead to adverse effects and withdrawal phenomena."**	Insomnia, cancer-related insomnia, sleep disorders	Ramakrishnan K, Scheid DC. Treatment options for insomnia. Am Fam Physician. 2007;76(4):517-526.<br><br>Sateia MJ, Buysse DJ, Krystal AD, Neubauer DN, Heald JL. Clinical practice guideline for the pharmacologic treatment of chronic insomnia in adults: an American Academy of Sleep Medicine clinical practice guideline. J Clin Sleep Med. 2017;13(2):307–349.	No	Recommend a review of all medications being taken; perhaps a blanket statement about discussing with your doctor. However do not include pharmacologic measures in our handout.
	**Melatonin:**<br>**"effective in patients with circadian rhythm sleep disorders and is safe when used in the short term"**	Circadian sleep disorders, ICU/critically ill patients, shift workers, jet-lag, insomnia, cancer-related insomnia	Ramakrishnan K, Scheid DC. Treatment options for insomnia. Am Fam Physician. 2007;76(4):517-526.<br><br>Pulak LM, Jensen L. Sleep in the Intensive Care Unit: A review. J Intensive Care Med. 2016;31(1):14-23. doi:10.1177/0885066614538749<br><br>Sateia MJ, Buysse DJ, Krystal AD, Neubauer DN, Heald JL. Clinical practice guideline for the pharmacologic treatment of chronic insomnia in adults: an American Academy of Sleep Medicine clinical practice guideline. J Clin Sleep Med. 2017;13(2):307–349.	Yes	Do not endorse melatonin use, but at least mention that melatonin may help with sleep since it is over the counter, but likely only short term. We see a lot of patients report melatonin use. May be useful for sleep onset but not for maintenance
	**Antidepressants:**<br>**"Effective treatment option in patients with insomnia and coexisting depression."**	Insomnia + depression	Ramakrishnan K, Scheid DC. Treatment options for insomnia. Am Fam Physician. 2007;76(4):517-526.<br><br>Sateia MJ, Buysse DJ, Krystal AD, Neubauer DN, Heald JL. Clinical practice guideline for the pharmacologic treatment of chronic insomnia in adults: an American Academy of Sleep Medicine clinical practice guideline. J Clin Sleep Med. 2017;13(2):307–349.	No	Do not mention prescription medication
	**Agomelatine:**<br>**"non-sedative antidepressant drug with agonistic action at melatonergic MT1 and MT2 receptors and antagonistic action at serotonergic 5-HT2c receptors, which can be a good choice for depressed patients with comorbid insomnia symptoms"**	Insomnia + depression	Fang H, Tu S, Sheng J, Shao A. Depression in sleep disturbance: A review on a bidirectional relationship, mechanisms and treatment. J Cell Mol Med. 2019 Apr;23(4):2324-2332. doi: 10.1111/jcmm.14170. Epub 2019 Feb 7. PMID: 30734486; PMCID: PMC6433686.<br><br>Hickie IB, Rogers NL. Novel melatonin-based therapies: potential advances in the treatment of major depression. Lancet. 2011 Aug 13;378(9791):621-31. doi: 10.1016/S0140-6736(11)60095-0. Epub 2011 May 17. PMID: 21596429.	No	Do not mention prescription medication
	**Nonbenzodiazepine hypnotics:**<br>**"effective treatments for chronic insomnia and, based on indirect comparisons, appear to have fewer adverse effects than benzodiazepines."**	Insomnia, cancer-related insomnia, sleep disorders	Ramakrishnan K, Scheid DC. Treatment options for insomnia. Am Fam Physician. 2007;76(4):517-526.<br><br>Sateia MJ, Buysse DJ, Krystal AD, Neubauer DN, Heald JL. Clinical practice guideline for the pharmacologic treatment of chronic insomnia in adults: an American Academy of Sleep Medicine clinical practice guideline. J Clin Sleep Med. 2017;13(2):307–349.	No	Do not mention prescription medication
**Pharmacologic therapy**<br><br>**(recommendations generally aimed at clinicians)**	**Antidiuretic: desmopressin acetate**<br>**"Medical therapies, such as desmopressin in patients with idiopathic nocturnal polyuria, can provide effective and safe relief for patients with persistent bother."**	Nocturnal polyuria (NP)	Weiss JP, Everaert K. Management of Nocturia and Nocturnal Polyuria. Urology. 2019;133S:24-33. doi:10.1016/j.urology.2019.09.022	No	Do not mention prescription medication
	"Medications for narcolepsy can include, sodium oxybate (Xyrem), stimulants, antidepressants, and selective serotonin reuptake inhibitors (SSRIs) and serotonin/norepinephrine reuptake inhibitors (SNRIs)"	Narcolepsy	Division of Sleep at Harvard Medical School via Wake Up Narcolepsy	No	Do not mention prescription medication
	"Armodafinil and modafinil are FDA-approved agents to reduce sleepiness associated with shift work"	Shift workers, narcolepsy	AASM	No	Do not mention prescription medication

	**Prescribed sleep aids for insomnia & cancer:<br>"Some sleep aids your doctor might recommend or prescribe include:<br><br>Zolpidem, Zolpidem CR (Ambien and other brands)<br>Eszopiclone (Lunesta)<br>Ramelteon (Rozerem, melatonin agonist)<br>Temazepam (Restoril)"**	Insomnia + cancer	American Cancer Society	No	Do not mention prescription medication
	**Prescription medications for RLS:<br><br>"common medicines your doctor might recommend or prescribe for RLS include:<br><br>Iron<br>Gabapentin (Neurontin and other brands) or gabapentin enacarbil (Horizant)<br>Different types of pain relievers or opioids<br>Clonazepam (Klonopin)"**	Restless Legs Syndrome	American Cancer Society	No	Do not mention prescription medication
	**Tricyclic antidepressants<br>"Sedating antidepressants are considered first-line agents when insomnia is comorbid with depression/anxiety symptomatology. These drugs include tricyclic antidepressants"**	Chronic pain, insomnia, depression, cancer	NIH National Cancer Institute	No	Do not mention prescription medication
	**Antihistamines with sedative effects<br>"Antihistamines are sold over the counter and are useful for treating difficulties in falling asleep only. There is limited evidence for the use of antihistamines to treat insomnia; these agents are used when traditional hypnotics or benzodiazepines are less suitable because of the risk of cross-dependence or other issues, such as vulnerability of a patient to addictions."**	Sleep disorders	NIH National Cancer Institute	No	Do not mention prescription medication
	**Antipsychotics with sedative effects<br>"have sedating effects caused mainly by the blockade of histamine receptors. However, these agents should be considered as a last resort and as a short-term treatment because of their serious side-effect profile"**	Sleep disorders	NIH National Cancer Institute	No	Do not mention prescription medication
	**Pitolisant<br>"We recommend that clinicians use pitolisant for the treatment of narcolepsy in adults" (AASM)**	Narcolepsy	Maski K, Trotti LM, Kotagal S, et al. Treatment of central disorders of hypersomnolence: an American Academy of Sleep Medicine clinical practice guideline. J Clin Sleep Med. 2021;17(9):1881–1893.	No	Do not mention prescription medication
	**Sodium oxybate<br>"We recommend that clinicians use sodium oxybate for the treatment of narcolepsy in adults" (AASM)**	Narcolepsy (adults)	Maski K, Trotti LM, Kotagal S, et al. Treatment of central disorders of hypersomnolence: an American Academy of Sleep Medicine clinical practice guideline. J Clin Sleep Med. 2021;17(9):1881–1893.	No	Do not mention prescription medication
	**Solriamfetol<br>"We recommend that clinicians use solriamfetol for the treatment of narcolepsy in adults" (AASM)**	Narcolepsy (adults)	Maski K, Trotti LM, Kotagal S, et al. Treatment of central disorders of hypersomnolence: an American Academy of Sleep Medicine clinical practice guideline. J Clin Sleep Med. 2021;17(9):1881–1893.	No	Do not mention prescription medication
	**Atypical antipsychotic medications<br>"The following therapies may be used for the treatment of PTSD-associated nightmares: ... the atypical antipsychotics olanzapine, risperidone and aripiprazole"**	PTSD associated nightmares, nightmare disorders	Morgenthaler TI, Auerbach S, Casey KR, et al. Position Paper for the Treatment of Nightmare Disorder in Adults: An American Academy of Sleep Medicine Position Paper. J Clin Sleep Med. 2018;14(6):1041-1055. Published 2018 Jun 15. doi:10.5664/jcsm.7178	No	Do not mention prescription medication
	**Relaxation therapy:<br>"Tensing and relaxing different muscle groups; biofeedback or imagery (visual and auditory feedback) to reduce somatic arousal; meditation; hypnosis "**	Insomnia	Ramakrishnan K, Scheid DC. Treatment options for insomnia. Am Fam Physician. 2007;76(4):517-526.<br><br>Edinger JD, Arnedt JT, Bertisch SM, et al. Behavioral and psychological treatments for chronic insomnia disorder in adults: an American Academy of Sleep Medicine systematic review, meta-analysis, and GRADE assessment. J Clin Sleep Med. 2021;17(2):263-298. doi:10.5664/jcsm.8988	Yes	Relevant and can be a helpful suggestion to promote sleep onset and/or what to do when you awake in the middle of the night and need to get back to sleep. Perhaps group under CBT.
	**Biofeedback therapy<br>"A variant of relaxation training that employs a device capable of monitoring and providing ongoing feedback on some aspect of the patient's physiology"**	Insomnia	Edinger JD, Arnedt JT, Bertisch SM, et al. Behavioral and psychological treatments for chronic insomnia disorder in adults: an American Academy of Sleep Medicine systematic review, meta-analysis, and GRADE assessment. J Clin Sleep Med. 2021;17(2):263-298. doi:10.5664/jcsm.8988	No	Relaxation therapy mentioned above should be sufficient.
	**Music therapy with a library of recorded musical selections<br>"The characteristics of music best suited for sleep and relaxation promotion are a tempo of approximately 60 beats/min, are composed primarily of low tones, and are played predominantly by strings"**	Post-op cardiac surgery pts	Pulak LM, Jensen L. Sleep in the Intensive Care Unit: A review. J Intensive Care Med. 2016;31(1):14-23. doi:10.1177/0885066614538749<br><br>Zimmerman L, Nieveen J, Barnason S, Schmaderer M. The effects of music interventions on postoperative pain and sleep in coronary artery bypass graft (CABG) patients. Sch Inq Nurs Pract. 1996;10(2):153-174.	Yes	Yes, can combine with relaxation therapy.

**Physical relaxation or comfort**	**Aromatherapy**<br>**"A combination of aroma, treatment and therapy, aromatherapy uses the aroma of essential oils to calm the mind, body, and spirit and achieve balance"**	ICU, critically ill	Jun J, Kapella MC, Hershberger PE. Non-pharmacological sleep interventions for adult patients in intensive care Units: A systematic review. Intensive Crit Care Nurs. 2021;67:103124. doi:10.1016/j.iccn.2021.103124<br><br>Cho EH, Lee MY, Hur MH. The Effects of Aromatherapy on Intensive Care Unit Patients' Stress and Sleep Quality: A Nonrandomised Controlled Trial. Evid Based Complement Alternat Med. 2017;2017:2856592. doi:10.1155/2017/2856592	Yes	Yes, can combine with relaxation therapy.
	**Back massage, acupressure**<br>**"Massage can improve sleep quality by increasing muscle relaxation, blood circulation and comfort."**	ICU, critically ill, hospitalized, cancer patients	Jun J, Kapella MC, Hershberger PE. Non-pharmacological sleep interventions for adult patients in intensive care Units: A systematic review. Intensive Crit Care Nurs. 2021;67:103124. doi:10.1016/j.iccn.2021.103124<br><br>Hsu WC, Guo SE, Chang CH. Back massage intervention for improving health and sleep quality among intensive care unit patients. Nurs Crit Care. 2019;24(5):313-319. doi:10.1111/nicc.12428<br><br>NIH National Cancer Institute (https://www.cancer.gov/about-cancer/treatment/side-effects/sleep-disorders-pdq#_26)	Yes	Relevant but not entirely practical, especially for patients with ostomies.
	**"Dress in loose, soft clothing."**	Hospitalized, cancer	NIH National Cancer Institute	Yes	Briefly include with relaxation.
	**"Changing sleep posture: Airway blockages are more likely when you sleep on your back, so switching to another sleeping position may help."**	Sleep apnea	SleepApnea.org	No	Remove as sleep posture is limited in this population.
**Devices, appliances**	**Oral appliance therapy:**<br>**"alternative to CPAP treatment for many people with obstructive sleep apnea. An oral appliance is a specially fitted device that you wear while sleeping. It helps position your mouth in a way that keeps your airway open."**	Obstructive sleep apnea	SleepApnea.org	No	Remove as low sleep apnea in this population.
	**Positive Air Pressure (PAP) therapy:**<br>**"Continuous positive airway pressure (CPAP) therapy is one of the most common ways of treating sleep apnea."**<br><br>**"Bilevel positive airway pressure (BiPAP or BPAP): two pressure levels, one for when a person inhales and another for when they exhale."**<br><br>**"Auto-titrating positive airway pressure (APAP): can automatically modify pressure levels in response to a person's breathing. In most cases, that pressure level is the same for both inhalation and exhalation."**	Obstructive sleep apnea	SleepApnea.org	No	Remove as low sleep apnea in this population.
	**Use your breathing device for all sleep, including naps."**	CPAP-wearing sleep apnea patients	NHLBI	No	Remove as low sleep apnea in this population.
	**Gradually ramping up CPAP pressure:**<br>**"A variety of technological advances may improve CPAP comfort and adherence, such as ramp-up features that allow the machine to start off at a low or minimal pressure as the patient adjusts and falls asleep"**	CPAP-wearing sleep apnea patients	Harvard Health	No	Remove as low sleep apnea in this population.
	**Bumper belt**<br>**"belts are designed to comfortably hold you on your side...when you try and roll over onto your back during sleep, these bumpers make it uncomfortable for you and could help to gradually train yourself to sleep on your side."**	CPAP-wearing sleep apnea patients	SleepAdvisor.org	No	Remove as low sleep apnea in this population.
	**Wear CPAP mask during the day:**<br>**"To help get used to wearing the mask during sleep, practice by wearing it during the day while sitting in a chair watching television or reading. This will distract your focus from the mask to a positive, familiar activity."**	CPAP-wearing sleep apnea patients	SleepApnea.org	No	Remove as low sleep apnea in this population.
	**"Consider a sleep-tracking device."**	Chronic kidney disease; dialysis	Renal Support Network (RSN)	Yes	Upon mentioning sleep diaries, aadd that sleep devices can be used to track sleep behaviors but only in the context of making sure that they keep a consistent sleep/wake schedule rather than to quantify exact sleep duration which may be inaccurate using these devices.
	**"Tape or PD catheter belts can make dressing with a catheter easy"**	Chronic kidney disease; home peritoneal dialysis patients	Renal Support Network (RSN)	No	Not relevant to HPN patients especially with the wording "catheter".
	**"Tape the fistula or graft needles securely in place using a technique called the chevron method"**	Chronic kidney disease; nocturnal hemodialysis	American Association of Kidney Patients (AAKP)	No	Irrelevant.
	**"Use enuresis pads (pads that absorb fluid), which alarm if a fluid/blood leak due to a disconnect occurs."**	Chronic kidney disease; nocturnal hemodialysis	American Association of Kidney Patients (AAKP)	No	Irrelevant.

Supplementary Table 2. Usefulness of 37 Sleep Recommendations Tailored for Home Parenteral Nutrition Consumers

Recommendation	Usefulness	
**1) Aim for 7 to 9 hours of sleep.**<br>A total of 7 to 9 hours of sleep is the recommended duration for most adults; however, you may need more or fewer hours of sleep. Keep in mind that this time reflects the time you spend asleep, and not the time you spend in bed. You may need to spend more time in bed to get 7 to 9 hours of sleep. This duration also does not need to be continuous or uninterrupted. Ideally your sleep will be of high quality and continuous, but it is not unusual to wake up several times in the middle of the night with TPN. If you are struggling with waking up too frequently or insomnia, discuss this with your healthcare team or seek a sleep specialist.	**Useful**	**9**
	Very Useful	3
	Somewhat Useful	6
	**Unuseful**	**1**
	Somewhat Unuseful	1
**2) Go to sleep and wake up at roughly the same time each day, even on weekends, days when you are off work, and on nights when you may not be running your TPN.**<br>A regular sleep schedule helps regulate your biological clock by signaling to your body when it is time to sleep. Having consistent sleep and wake times helps you fall asleep and wake up when you want to.	**Useful**	**10**
	Very Useful	3
	Somewhat Useful	7
	**Unuseful**	**0**
	Somewhat Unuseful	0
**3) Maintain a consistent TPN infusion schedule by starting your TPN at the same time on days when you run your TPN.**<br>Consistent TPN infusion times may also help your body better prepare for sleep. However, it might be stressful to have a fixed TPN schedule. Discuss the possibility of a flexible TPN schedule with your healthcare team.	**Useful**	**10**
	Very Useful	7
	Somewhat Useful	3
	**Unuseful**	**0**
	Somewhat Unuseful	0
**4) Do not go to bed unless you are ready to fall asleep.**<br>It is best to wait until you are ready to fall asleep before going to bed. This will help you avoid lying awake in bed for too long, which may be frustrating. If you think that you may be struggling with insomnia, talk to your healthcare team or a sleep specialist about other options.	**Useful**	**9**
	Very Useful	4
	Somewhat Useful	5
	**Unuseful**	**1**
	Somewhat Unuseful	1
**5) Avoid spending too much time in bed.**<br>Use your bed only for sleep and intimacy. There may be exceptions if you are feeling unwell or recovering from surgery.	**Useful**	**9**
	Very Useful	3
	Somewhat Useful	6
	**Unuseful**	**1**
	Somewhat Unuseful	1
**6) If you are unable to fall asleep after 20 minutes of being in bed, get out of bed instead of forcing yourself to sleep.**<br>Engaging in a relaxing activity outside of bed may help you feel sleepy. Return to bed when you are sleepy again.	**Useful**	**7**
	Very Useful	3
	Somewhat Useful	4
	**Unuseful**	**3**
	Somewhat Unuseful	3
**7) Create a relaxing bedtime routine.**<br>This can include reading, journaling, or some other calming activity.	**Useful**	**9**
	Very Useful	4
	Somewhat Useful	5
	**Unuseful**	**1**
	Somewhat Unuseful	1
**8) Avoid engaging in stimulating or stressful activities in the evening.**<br>Avoid activities that you find stressful before bed, such as watching the news or checking your email. Some useful ways to manage stress before bed include journaling, writing down thoughts that are on your mind, getting organized, and setting goals.	**Useful**	**10**
	Very Useful	7
	Somewhat Useful	3
	**Unuseful**	**0**
	Somewhat Unuseful	0
**9) Limit exposure to bright light in the evenings, including bright light from electronic devices.**<br>Limiting light exposure tells your body that it is time to sleep. Try to limit the use of electronic devices, including phones and TV, to at least 30 minutes before your bedtime. Using dimly lit lamps or light dimme switches can help limit your exposure to bright light in the evenings. If your TPN pump flashes often, consider covering it. Also, changing the lighting settings of your electronic devices to warmer tones ("Night Shift Mode") or wearing blue-light-blocking glasses before bed can help limit your exposure to bright light.	**Useful**	**10**
	Very Useful	9
	Somewhat Useful	1
	**Unuseful**	**0**
	Somewhat Unuseful	0
**10) Empty your bladder, bowel, and ostomy bag before going to bed.**<br>Voiding right before going to bed can help limit the number of bathroom trips you make at night.	**Useful**	**9**
	Very Useful	5
	Somewhat Useful	4
	**Unuseful**	**1**
	Somewhat Unuseful	1

11.) If safe and possible, limit your fluid intake before bedtime.
Limiting your fluid intake before bed can help reduce the number of bathroom trips you make at night. Reducing your overall fluids and IV hydration or changing the time to daytime may help you sleep better. Always discuss your fluid intake and IV hydration with your healthcare team before making any changes.

**Useful**	**7**
Very Useful	3
Somewhat Useful	4
**Unuseful**	**3**
Somewhat Unuseful	3

12.) If you eat food by mouth, consider earlier mealtimes, and avoid caffeine and alcohol before bed.
Aim to stop eating at least 3 hours before going to bed. This can help you fall asleep and stay asleep more easily. Avoid both caffeine and alcohol in the evening as they make it more difficult for you to fall asleep and stay asleep. However, if you eat by mouth, a light snack is okay if you are feeling hungry at

**Useful**	**7**
Very Useful	3
Somewhat Useful	4
**Unuseful**	**3**
Somewhat Unuseful	3

13.) Keep your bedroom cool while you sleep.
An excessively hot or cold room may make it harder for you to fall asleep and stay asleep. Aim for a bedroom that is between 60 to 68 degrees Fahrenheit or 16 to 20 degrees Celsius.

**Useful**	**10**
Very Useful	6
Somewhat Useful	4
**Unuseful**	**0**
Somewhat Unuseful	0

14.) Keep your bedroom as dark as safely possible.
Having a dark bedroom helps you fall asleep and stay asleep. However, if you need some light in your bedroom or hallway, keep it as dim as possible and facing away from your face. For example, floor lights can help keep your bedroom dimly lit without disrupting your sleep.

**Useful**	**9**
Very Useful	5
Somewhat Useful	4
**Unuseful**	**1**
Somewhat Unuseful	1

15.) Make sure the area around your bed is clutter-free.
You may need to wake up in the middle of the night to use the bathroom or troubleshoot your pump. Creating a clutter-free space around your bed can make this quicker and safer so that you can get right back to bed.

**Useful**	**10**
Very Useful	6
Somewhat Useful	4
**Unuseful**	**0**
Somewhat Unuseful	0

16.) Make sure your path to the bathroom is clear in case you need to use the bathroom at night.
Using the bathroom at night is often necessary. Find ways to make trips to the bathroom at night quicker and safer so that you can get right back to bed. For example, use a cart or trolley bag to help you move your pump, remove any rug or carpet that may make you trip, use floor lighting and dimly lit hallways, install handrails to prevent you from falling, or move your bed closer to the bathroom.

**Useful**	**10**
Very Useful	6
Somewhat Useful	4
**Unuseful**	**0**
Somewhat Unuseful	0

17.) A bedside commode can make bathroom trips quicker and easier.
Although not for everyone, if getting to the bathroom is difficult for you, a bedside commode can sometimes help.

**Useful**	**6**
Very Useful	2
Somewhat Useful	4
**Unuseful**	**4**
Somewhat Unuseful	4

18.) Keep your bedroom as quiet as possible.
If you find that your pump alarms are loud and disruptive, consider safely masking the noise. Some ways to do this include covering the pump, placing the pump in a pillowcase or bag, or using a white noise machine. However, you should still be able to hear important emergency alarms. If you use multiple pumps, try to synchronize the pumps to limit the number of alarms at night. You should not have to deal with many alarms during the night; try to find out what is causing the alarms to ring at night. If you are stil struggling with too many alarms at night, speak to your healthcare team about troubleshooting or switching to a newer pump.

**Useful**	**8**
Very Useful	5
Somewhat Useful	3
**Unuseful**	**2**
Somewhat Unuseful	1
Very Unuseful	1

19.) Prepare your bed area to maximize comfort while running TPN at night.
You may experience feeling hot or cold during TPN infusions, and preparing for this can help you sleep more comfortably. For example, if you often experience night sweats, sleeping on a towel can help you fall back asleep quicker. Having an extra blanket for night chills, bringing the TPN bag to room temperature before starting your infusion, or having extra cleaning supplies in case of leaks may help you during your sleep.

**Useful**	**9**
Very Useful	5
Somewhat Useful	4
**Unuseful**	**1**
Somewhat Unuseful	1

20.) If you have a bed partner, talk to them about your sleep needs while running your TPN.
Social support is important when receiving TPN at home, so involve your bed partner to get the help you need. For some people, it may be helpful to sleep in separate beds or rooms to avoid disrupting the slee of others.

**Useful**	**8**
Very Useful	4
Somewhat Useful	4
**Unuseful**	**2**
Somewhat Unuseful	2

21.) Go outside and get natural sunlight for at least 30 minutes each day.
Exposing yourself to sunlight, especially in the morning, can help you feel awake and refreshed.

**Useful**	**10**
Very Useful	3

	Somewhat Useful	7
	**Unuseful**	**0**
	Somewhat Unuseful	0
**22.) Try to stay active during the day.**<br>Walking, exercising, and staying active during the day can improve your sleep. Talk to your healthcare team about being active during the day.	**Useful**	**8**
	Very Useful	1
	Somewhat Useful	7
	**Unuseful**	**2**
	Somewhat Unuseful	2
**23.) Limit naps whenever possible.**<br>Limiting naps during the day can be challenging especially if you did not get a good night's sleep. If you need to take a nap, try to keep your nap to 30 minutes or less and before 3pm so that you can fall asleep at night. However, more frequent napping may be necessary when you are feeling unwell or recovering from surgery.	**Useful**	**7**
	Very Useful	2
	Somewhat Useful	5
	**Unuseful**	**3**
	Somewhat Unuseful	2
	Very Unuseful	1
**24.) Try tracking your sleep for a couple of days using a sleep diary.**<br>Tracking your sleep for at least one week can help you and your healthcare team find ways to improve your sleep and identify behaviors that may cause you to sleep poorly. You can use our sleep diary below to track your sleep and TPN infusion. If you prefer to track your sleep electronically, you can also use a sleep tracking device and mobile application available for your phone. Keep in mind that sleep duration estimated by wearable technology and phone apps are not always accurate.	**Useful**	**10**
	Very Useful	5
	Somewhat Useful	5
	**Unuseful**	**0**
	Somewhat Unuseful	0
**25.) Discuss with your healthcare team making changes to your TPN infusion schedule.**<br>Longer infusion cycles, for example 12 hours instead of 10 hours, can help reduce the number of bathroom trips at night. Shorter infusion cycles, however, can help you run your TPN quicker. Starting infusions earlier in the evening may also help by having the infusions end earlier in the morning. Discuss these possibilities, including changes to your IV hydration or fluids, with your healthcare team before making any changes.	**Useful**	**9**
	Very Useful	6
	Somewhat Useful	3
	**Unuseful**	**1**
	Somewhat Unuseful	1
**26.) Running your TPN during the daytime can help you sleep better.**<br>TPN during the daytime may be inconvenient for many. If you find that daytime TPN is too difficult, you can consider doing some days of TPN during the day and others during the night. Running your fluids and IV hydration during the day may also help.	**Useful**	**8**
	Very Useful	5
	Somewhat Useful	3
	**Unuseful**	**2**
	Somewhat Unuseful	1
	Very Unuseful	1
**27.) Consider a different sleeping posture if your TPN at night is causing you discomfort.**<br>Sleeping inclined may help relieve indigestion, reflux, and nausea.	**Useful**	**8**
	Very Useful	3
	Somewhat Useful	5
	**Unuseful**	**2**
	Somewhat Unuseful	2
**28.) If you struggle with line tangling and mispositioning, or fear line dislodgement while sleeping, consider longer line tubing or try securing your line.**<br>Longer line tubing can give you more wiggle room to shift positions at night. Using central line wraps or PICC line sleeves can also help secure lines.	**Useful**	**10**
	Very Useful	7
	Somewhat Useful	3
	**Unuseful**	**0**
	Somewhat Unuseful	0
**29.) Consider replacing your IV pole with a backpack for easier movement at night.**<br>Hanging your TPN bag on an IV pole may make it difficult for you to move at night, especially on a carpet or rug. Try using a cart or trolley bag to help you move your pump around.	**Useful**	**10**
	Very Useful	7
	Somewhat Useful	3
	**Unuseful**	**0**
	Somewhat Unuseful	0
**30.) Review your list of medications and fluids with your healthcare team for possible sleep interference.**<br>Some medications and getting too much IV hydration may affect how you sleep. Review your medications and the amount of fluids you are receiving with your healthcare team to find which ones may be disrupting your sleep.	**Useful**	**9**
	Very Useful	7
	Somewhat Useful	2
	**Unuseful**	**1**
	Somewhat Unuseful	0
	Very Unuseful	1
**31.) If you smoke tobacco or vape, try quitting.**<br>Nicotine can make it difficult for you to fall asleep and stay asleep.	**Useful**	**6**
	Very Useful	4
	Somewhat Useful	2
	**Unuseful**	**4**

	Somewhat Unuseful	0
	Very Unuseful	4
**32.) Consider the following sleep aids that may help you with your sleep: eye masks, earplugs, blackout curtains, weighted blankets, and white noise machines.** Different aids work well for some people. Test different aids for several days at a time to find out which ones may help you fall asleep and stay asleep.	**Useful**	**10**
	Very Useful	5
	Somewhat Useful	5
	**Unuseful**	**0**
	Somewhat Unuseful	0
**33.) Create new relaxing behaviors before bed.** This can include listening to calming music or nature sounds, practicing yoga, relaxing your muscles to relieve tension, wearing loose and soft clothing, and aromatherapy using essential oils.	**Useful**	**10**
	Very Useful	4
	Somewhat Useful	6
	**Unuseful**	**0**
	Somewhat Unuseful	0
**34.) Practice meditation and mindfulness to help you relax and reduce stress before going to bed.** Guided meditation available on different mobile applications can help you learn new ways to control your thoughts and breathing. For example, one technique is guided imagery, in which you visualize positive images or scenarios in your mind to help your body relax and relieve stress and anxiety. You can find free guided meditation resources and mobile applications on the internet.	**Useful**	**10**
	Very Useful	5
	Somewhat Useful	5
	**Unuseful**	**0**
	Somewhat Unuseful	0
**35.) Cognitive Behavioral Therapy for Insomnia (CBT-I)** CBT-I is a common, safe, and effective long-term treatment for people with insomnia that may be helpful if you are struggling with getting to sleep or staying asleep. Cognitive therapy, Sleep restriction therapy, Stimulus control therapy, Sleep hygiene, and Relaxation techniques are forms of CBT-I. CBT-I needs to be administered by a clinician, so please discuss this therapy with your healthcare team. You can find a trained clinician in CBT-I on the internet.	**Useful**	**9**
	Very Useful	7
	Somewhat Useful	2
	**Unuseful**	**1**
	Somewhat Unuseful	1
**36.) Melatonin may help you fall asleep for a few nights but is unlikely to help in the long term.** Sleep medications may be useful for a few nights. Discuss sleep medications and supplements with your healthcare team before taking them.	**Useful**	**9**
	Very Useful	4
	Somewhat Useful	5
	**Unuseful**	**1**
	Somewhat Unuseful	1
**37.) If your sleep problems continue, consider discussing other options with your healthcare team.** Please contact your healthcare team or a sleep specialist to rule out sleep disorders such as insomnia, sleep apnea, and restless legs syndrome.	**Useful**	**10**
	Very Useful	5
	Somewhat Useful	5
	**Unuseful**	**0**
	Somewhat Unuseful	0
